# An aggregation-prone region in talin controls talin self-interactions and regulates integrin adhesion complex dynamics

**DOI:** 10.1073/pnas.2602326123

**Published:** 2026-11-04

**Authors:** Neil J. Ball, Paula Turkki, Yanyu Guo, Mahmudul H. Tanvir, Shimin Le, Jasmine Fei Li Chin, Karen B. Baker, Jingxing Luo, Paul Atherton, Nicholas H. Brown, Vesa P. Hytönen, Jie Yan, Benjamin T. Goult

**Affiliations:** ^a^ Department of Biochemistry, Cell and Systems Biology, Institute of Systems, Molecular and Integrative Biology, University of Liverpool, Liverpool L69 7ZB, United Kingdom; ^b^ Faculty of Medicine and Health Technology, Tampere University, Tampere 33520, Finland; ^c^Fimlab Laboratories, Tampere 33520, Finland; ^d^ Department of Physics, National University of Singapore, Singapore 117542, Singapore; ^e^ Mechanobiology Institute, National University of Singapore, Singapore 117411, Singapore; ^f^ School of Biosciences, University of Kent, Canterbury CT2 7NJ, Kent, United Kingdom; ^g^ Department of Molecular and Clinical Cancer Medicine, Institute of Systems, Molecular and Integrative Biology, University of Liverpool, Liverpool L69 3GE, United Kingdom; ^h^ Department of Physiology, Development and Neuroscience, University of Cambridge, Cambridge CB2 1DY, United Kingdom; ^i^ Liverpool Interdisciplinary Neuroscience Centre, University of Liverpool, Liverpool L9 7AL, United Kingdom

**Keywords:** mechanobiology, integrin, cell adhesion

## Abstract

Talin is a mechanosensitive scaffold that couples integrins to the actin cytoskeleton and coordinates signaling at integrin adhesion complexes. A second integrin-binding site (IBS2) has been proposed within the R11–R12 region of the talin rod, although the molecular basis of this interaction has remained unclear. Here, we show that talin R11 contains a cryptic aggregation-prone region (APR) embedded within Helix50. Mechanical unfolding of R11 exposes this motif, enabling high-affinity talin–talin interactions and force-dependent self-association. The isolated APR forms amyloid-like fibers in vitro, and APR-disrupting gatekeeper mutations suppress fiber formation, talin self-association, and cellular recruitment. We therefore designate this motif Aggregation-Prone Region 1 (APR1). Mitochondrial-targeting experiments demonstrate that APR1-dependent recruitment occurs in cells independently of focal adhesions and integrins. APR1 overlaps the vinculin-binding site in Helix50, and vinculin disrupts preexisting talin assemblies, revealing a mechano-chaperone function for vinculin. Disruption of APR1-mediated self-association does not impair adhesion formation but instead generates larger, more talin-rich adhesions with altered assembly dynamics and signaling properties, indicating a role for APR1 in regulating adhesion remodeling. Together, these findings identify force-dependent talin self-association as a mechanism in mechanotransduction and suggest that the region previously designated IBS2 functions primarily as an aggregation-prone regulatory element.

## Significance

Cells sense and respond to mechanical forces through integrin adhesion complexes. Talin is a central mechanosensitive scaffold within these structures, containing a series of force-sensitive switch domains that control signaling. Here, we show that mechanical unfolding of talin R11 exposes a cryptic aggregation-prone region that drives force-dependent talin self-association. Rather than simply recruiting additional talin molecules, disruption of this mechanism produces larger, more talin-rich adhesions with altered assembly dynamics and signaling, revealing an unexpected role in adhesion remodeling. We further identify vinculin as a mechano-chaperone that suppresses talin self-association by competing for the same mechanically exposed site. These findings reveal a mechanism by which cells interpret mechanical information and regulate adhesion dynamics.

The adhesive structures that cells form to attach to the extracellular matrix (ECM) are beautiful in their sophistication and complexity, able to form numerous different signaling complexes and structures as a function of the properties of the ECM. The integrin family of ECM-receptors mediate this adhesion and large integrin adhesion complexes (IACs) assemble at these sites, coupling the ECM to the cytoskeleton via the adaptor protein talin. A significant advance has been the realization that, despite their structural complexity [containing potentially hundreds of proteins (1)], these adhesion complexes contain a simple and robust core of just four proteins: integrin, talin, kindlin, and actin (2, 3).

Talin is a large multidomain protein, that comprises an N-terminal FERM domain (with subdomains F0–F3), followed by an ~80 aa unstructured linker, and then 13 helical bundles, R1–R13 and a dimerization helix (DD), which form the talin rod (4, 5). Each rod domain has the potential to function as a mechanochemical switch, as it can reversibly switch between folded “0” and unfolded “1” states in response to mechanical forces (5–8). These different states can recruit different proteins to alter IAC composition (9, 10). Many talin-interacting proteins have been identified (2), emphasizing the ability of talin to function as a mechanosensitive signaling hub (11).

Forming a mechanical linkage between the ECM–integrin complex and actin involves direct interactions between talin, integrin, and actin. The binding of talin to integrin can potentially be mediated by either of two reported integrin-binding sites (IBSs) (12–14). The direct binding of IBS1 to the cytoplasmic tail of the integrin beta subunit has been well established by numerous experimental approaches (reviewed in ref. 12), including atomic structures of the complex (15), however for IBS2, the evidence is less clear-cut. The presence of an integrin binding site in the rod domain was first described as integrin binding to the two talin fragments formed by calpain cleavage (head and rod) (16). Although the data for the rod binding integrin were not shown it suggested that there were at least two integrin-binding sites in talin. This was followed by demonstration that a region of the talin rod (residues 1984–2541; comprising part of R11, R12, and R13) bound to integrin β3 coated wells (17). A region of the talin rod that was sufficient for localization of rod fragments to focal adhesions (FAs) was mapped to a 130-residue region, and then refined to a 23 residue fragment (residues 2077–2099), corresponding to helix 50 of R11 (18). The structure of R11–R12 showed two 5-helix bundles sharing a long alpha helix, and both R11–R12 were found to be required for integrin binding (19). A common feature of the biochemical assays used to demonstrate IBS2 binding to integrin is that they all used a solid phase approach (17–19), with the integrin cytoplasmic domain attached to a surface. Binding of IBS2 to integrin in solution has yet to be reported.

An interaction between IBS2 and integrin has also been supported by genetic experiments in *Drosophila*. Point mutations in either IBS1 or IBS2 impair talin function, and the double mutant is stronger, indicating some compensatory function between the two IBSs (20). Analysis of a large number of truncation mutants of talin, revealed that Helix50 was particularly important in mediating adhesion between epithelial layers, but not for mediating muscle attachment to tendon matrix (21). Using FRET, integrin and IBS2 were found in close proximity in the epithelium, but not the muscle, and the IBS2s in different talin molecules were found close together in vivo.

In this study, we report the identification of an aggregation-prone region (APR) in talin, which maps to Helix50 in R11. We show that the oligomeric status of R11 is very conformationally sensitive, and changes in force and/or redox conditions can expose this APR and trigger oligomerization. We show that Helix50 is involved in three different interactions, i) in the folded “0” state the R11 bundle is involved in binding to the ligands tensin-3 (22) and RIAM (5), ii) in the unfolded “1” state where the domain is unfolded to a string of helices, Helix50 binds vinculin (19, 23, 24), and iii) when unfolded to an unstructured polypeptide it forms a high affinity binding site for other Helix50s. We propose the name Aggregation-Prone Region 1 (APR1) for this talin oligomerization motif. In this way we suggest that there may not be a second integrin-binding site in the talin rod and instead the data supporting IBS2 binding to integrin can be reinterpreted as talin oligomerization. This leads to a new model in which unfolding of R11 exposes APR1, enabling force-dependent talin self-association that regulates adhesion remodeling and mechanosensitive signaling within mature FAs. Finally, we show that vinculin binding can resolve these R11 oligomers, thus revealing a role for vinculin as a mechano-chaperone.

## Results

### Talin R11 Can Form Soluble Higher-Order Species.

**Fig. 1.**
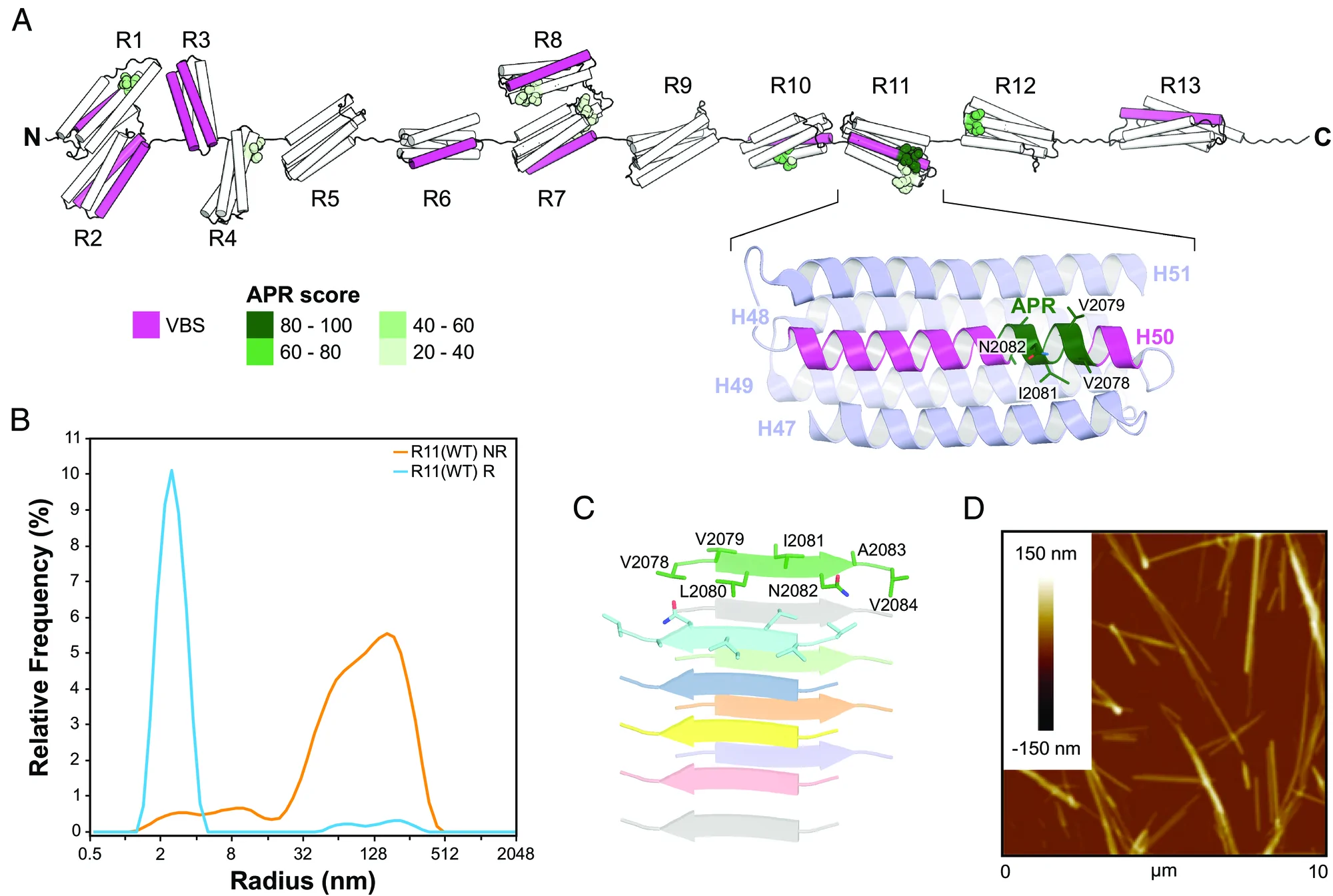
R11 contains an APR that can form amyloid-like fibrils in vitro. (*A*) Location of predicted APRs in the talin rod. Predicted APRs are shown as spheres and colored by APR score in shades of green, as calculated by the TANGO algorithm (25). Vinculin-binding sites (VBSs) are shown in magenta. The highest-scoring APR is located in R11 and corresponds to residues ^2078^VVLINAV^2084^ within Helix50. The zoomed view of R11 shows that APR1 overlaps with the VBS in Helix50. (*B*) Talin R11 can form a large higher-order species. DLS analysis of reduced (blue) and nonreduced (orange) R11. Nonreduced R11 contains a large species with a hydrodynamic radius of ~128 nm, whereas addition of reducing agent collapses this to a single species at ~2.47 nm, consistent with monomeric R11. (*C*) AlphaFold structural model of 10 copies of the ^2078^VVLINAV^2084^ APR region from R11. The APR is predicted to form β-strands that can self-assemble into stacked β-sheets. (*D*) Atomic Force Microscopy (AFM) image of a synthetic ^2078^VVLINAV^2084^ peptide showing self-assembly into extended micron-long amyloid-like fibrils.

Expression and purification of recombinant talin R11 (residues 1974–2140) gives rise to a well behaved soluble protein (19). However, we noticed that over time the protein can undergo changes in its properties. This can be visualized in *SI Appendix*, Fig. S1*A* where partially purified R11 protein was processed in two different ways prior to loading onto an anion exchange column. When immediately diluted with 20 mM Tris pH 8.0, 50 mM NaCl, a single peak is observed (light blue in *SI Appendix*, Fig. S1*A*), whereas after dialysis overnight into the same buffer the elution profile contains two peaks (dark blue in *SI Appendix*, Fig. S1*A*). Analysis of these two peaks by reducing SDS-PAGE revealed both peaks contain the same protein (*SI Appendix*, Fig. S1*B*). However, the use of nonreducing sample buffer revealed that the second peak is twice the size of the monomeric protein, suggesting that R11 forms a disulfide-bonded dimer (*SI Appendix*, Fig. S1*B*). R11 contains a single cysteine residue, Cys1978, that is at the start of the first helix (Helix47) and is buried in the core of the folded R11 domain (*SI Appendix*, Fig. S1*C*). The broad nature of the additional nonreduced ion exchange peak led us to examine it by Dynamic Light Scattering (DLS) to look at its hydrodynamic radius (Fig. 1*B*). DLS revealed that the nonreduced R11 sample has a very large hydrodynamic radius (~128 nm), which is dramatically larger than a dimer (Fig. 1*B*, orange). Addition of the reducing agents Dithiothreitol (DTT), or Tris(2-carboxyethyl)phosphine (TCEP; Fig. 1*B*, cyan) fully converted the large species back to the monomeric form, as analyzed by DLS and SDS-PAGE. The estimated molecular weight of a protein with hydrodynamic radius of 128 nm is 800 MDa, corresponding to a multimer with approximately 40,000 copies. This indicates that the single buried cysteine residue in R11 forms a disulfide bond between two R11 monomers and stabilizes an open conformation of R11 that can form large soluble higher-order species. Importantly, this species remained soluble and could be fully converted back to the monomeric form by addition of TCEP.

### Computational Analysis Identifies Multiple APRs in Talin.

The large species of R11 that can form once R11 is partially unfolded indicated that the domain contains a cryptic talin interaction motif. To look for this motif we used the TANGO software (25), which predicts APRs in proteins. These APRs have the propensity to form intermolecular beta-sheets and are established forms of protein oligomerization, best known for their role in pathogenic amyloid formation (26), but many examples of functional APR motifs have been reported. We note that the term “aggregation” is often incorrectly thought of as solely a negative behavior of ill-behaved proteins, but functional protein aggregation is a recognized phenomenon in many systems (25). TANGO analysis of talin-1 revealed multiple APRs distributed through the talin rod (Fig. 1*A*) and it was striking that these APRs are predominantly located buried within the folded talin rod domains (Fig. 1*A*). The highest-scoring APR corresponded to the sequence ^2078^VVLINAV^2084^ within Helix50 of talin-1 R11. This motif is conserved in talin-2, differing by only a conservative Val-to-Ile substitution within the core APR sequence (VVLINAV in talin-1; VVLINAI in talin-2). This motif is also highly conserved across vertebrate talins, including complete conservation of the VVLINAV sequence in zebrafish talin. This sequence is part of the 4th helix of the 5-helix bundle and forms part of the VBS in Helix50 (Fig. 1 *A*, *Inset*). The crystal structures of R11–R12 [PDB 3DYJ (19)] and Helix50 in complex with vinculin D1 domain (VD1) [PDB 4DJ9 (24)] have both been solved, showing that this APR region is an integral part of Helix50 both in the folded talin R11 bundle and when bound to VD1.

### AlphaFold Structural Modeling Indicates That the APR Region of R11 Can Form Intermolecular Beta-Sheet Structures.

To further explore the potential of this VVLINAV region to form oligomers we used AlphaFold3 (27) to generate a structural model of 10 copies of a VVLINAV peptide (Fig. 1*C*). The resultant structural model predicted the beta-sheet amyloid arrangement of the 10 talin peptides, with each VVLINAV motif forming a beta-strand that stacks into a parallel beta-sheet with these beta-sheets stacked together in an antiparallel orientation.

### The Isolated APR From R11 Forms Micron-Long Amyloid Fibers, Which Can Be Eliminated by a V2078K Gatekeeper Mutation.

To validate this predicted APR region, we synthesized two peptides, ^2078^VVLINAV^2084^, and a version where we introduced a “gatekeeper” residue, mutating V2078 to lysine, ^2078^KVLINAV^2084^. Gatekeeper residues are charged residues that are positioned at the start of an APR that suppress intermolecular β-sheet assembly through charge repulsion (28). We chose the first valine, Val2078, to mutate due to it being solvent exposed in the structure of R11 (Fig. 1*A*). The VVLINAV peptide readily formed fibrous structures which were visible to the naked eye. When visualized with AFM, often used to examine amyloid structures (29), these fibers were observed to be microns long and of consistent diameter (Fig. 1*D*). In contrast, introduction of the canonical gatekeeper mutation V2078K abolished fiber formation. The sensitivity of self-assembly to a single gatekeeper substitution supports the conclusion that this behavior is driven by a bona fide aggregation-prone sequence rather than nonspecific hydrophobic interactions.

### The Cysteine Is Not Required for R11 Self-Assembly If R11 Is Destabilized.

Both cysteine 1978 and the APR region in R11 are cryptic, buried in the folded R11 domain (Fig. 1*A* and *SI Appendix*, Fig. S1*C* respectively) and so are inaccessible to interactions without a conformational change. This suggested that factors that induce conformational changes in R11 may trigger oligomerization. The formation of the disulfide-mediated R11 dimer appears to be necessary for the formation of larger species of R11 in vitro, as these do not form in a reducing environment. This led us to test three hypotheses, i) the cysteine is required for big species formation, ii) R11 dimerization is required for big species formation and iii) it is the conformational change that is required for big species formation. To test these hypotheses, we made a series of new constructs of R11. First, we mutated the cysteine in R11 to alanine (C1978A). The R11(C1978A) protein behaved well and produced a monodisperse single species in DLS in both reducing and nonreducing conditions (*SI Appendix*, Fig. S1*D*) with a hydrodynamic radius equal to that of a monomer, identical to the reduced R11(WT) (Fig. 1*B*; light blue). This confirmed that in vitro the cysteine is necessary for large species formation of R11. Next, we wanted to test whether disulfide-mediated dimerization by itself is sufficient for aggregate formation, so we designed a further construct where we added a cysteine attached via a short linker to the C-terminus of R11(C1978A) (R11(C1978A)-Cys, *SI Appendix*, Fig. S1 *E*, *Inset*). R11(C1978A)-Cys readily formed dimers in nonreducing conditions (*SI Appendix*, Fig. S1*E*) but was unable to form large species, supporting the idea that it is not dimerization of the R11 itself but the disulfide-mediated stabilization of the unfolded species of R11 that is required. Finally, to test the requirement of conformational change to expose the APR motif, we made an R11 construct with a deletion of the cysteine-containing first helix (Helix47; residues 1974–2009). Deleting the first helix both removes the cysteine and destabilizes the domain. This truncated, R11(∆H47) protein readily formed large aggregates, even in reducing conditions (*SI Appendix*, Fig. S1*F*; dark blue). Taken together, these data point to R11 unfolding as the important trigger for self-assembly, a process accelerated by the disulfide-mediated stabilization of the unfolded state.

### Talin R11 Is Recruited to Cell Adhesions Formed by R11-Containing Talin.

**Fig. 2.**
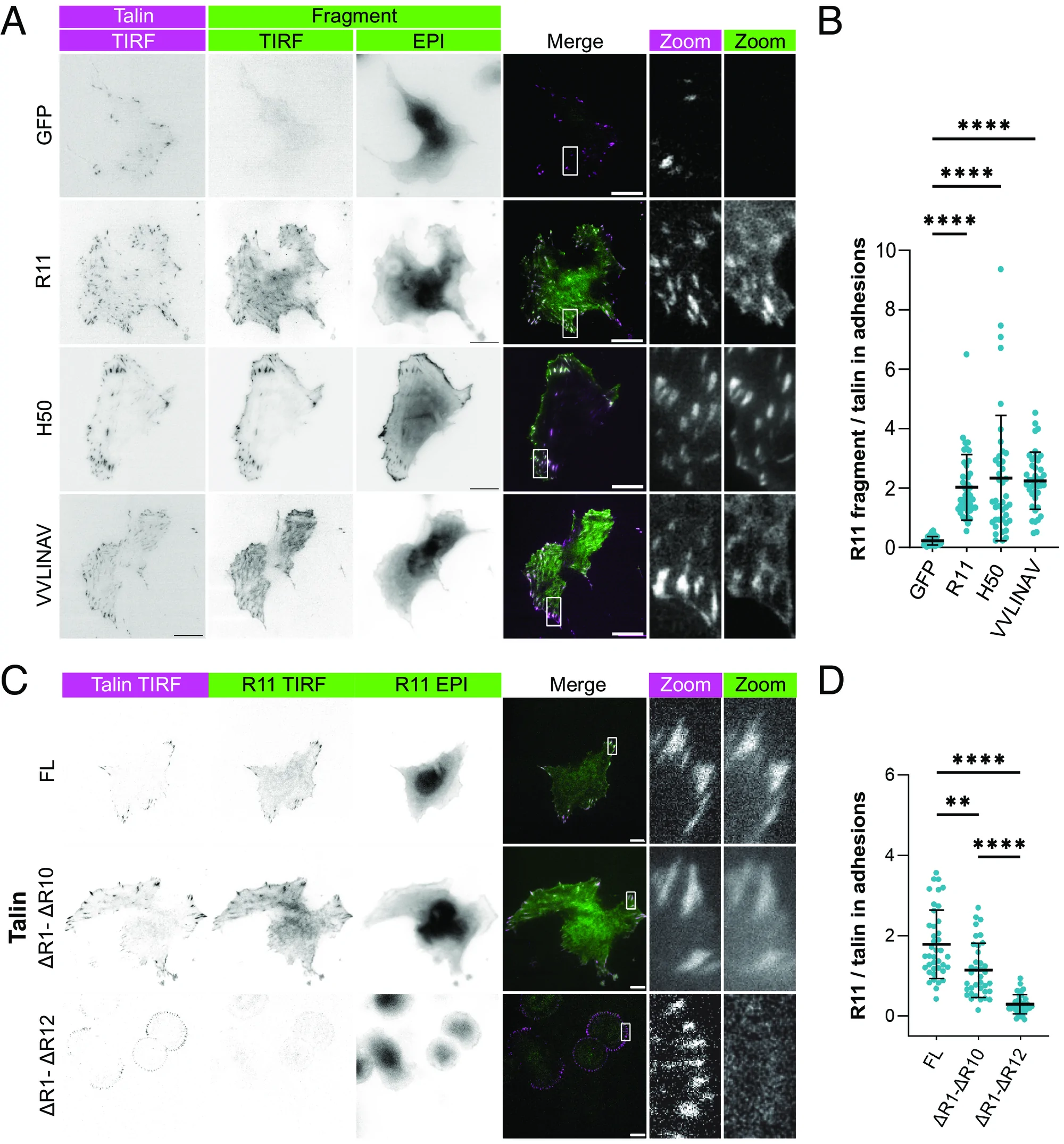
Recruitment of talin R11 to FAs requires talin with R11 to be already present. (*A*) Representative TIRF images showing colocalization of GFP-tagged R11 fragments (GFP-R11, GFP-H50, and GFP-VVLINAV) with mCherry full-length talin-1, talin(FL) at FAs. (Scale bar, 10 µm.) (*B*) Quantitative analysis of R11 fragment colocalization at adhesion sites with talin(FL) (two independent experiments n = 37 to 41 cells). Colocalization within adhesions was quantified as the GFP intensity ratio relative to mCherry intensity. (*C*) Representative TIRF images showing colocalization of the GFP-R11 with talin(FL) and talin lacking domains R1–R10, Talin(ΔR1–R10), but not with talin lacking domains R1–R12, Talin(ΔR1–R12). (Scale bar, 10 µm.) (*D*) Quantification of GFP-R11 colocalization at adhesion sites for the indicated talin constructs (three independent experiments n = 30 to 40 cells). A similar analysis for GFP-VVLINAV is shown in *SI Appendix*, Fig. S2. Statistical significance was assessed using Brown–Forsythe and Welch ANOVA tests. Data are presented as mean and SD from three independent biological replicates. Statistical significance is indicated as follows: *P < 0.05; **P < 0.01; ****P < 0.0001.

Having established that self-interactions of R11 occur in vitro, we next wanted to explore the role of R11–R11 interactions at FAs in cells. IACs require the presence of talin to mediate the mechanical linkage between the integrin–ECM complex and the cytoskeleton. Removal of talin results in cells rounding up and detaching (3) as exemplified in *Tln1^−/−^Tln2^−/−^* MKF cells that are round and nonadherent. Transfection of these cells with wild type talin constructs restores their ability to adhere and form FAs (Fig. 2*A*). This makes them ideal for looking at specific talin functions as they lack endogenous talin, which would otherwise complicate the analysis of modified talin constructs.

We generated three EGFP-tagged R11 constructs, namely GFP-R11, GFP-Helix50 (GFP-H50), and GFP-VVLINAV and coexpressed them, or GFP alone as a control, with mCherry/mScarlet-labeled full-length talin (Talin(FL)). Upon expression of Talin(FL) the cells spread and made FAs. Coexpression of GFP-R11, GFP-Helix50, and GFP-VVLINAV revealed that all three R11 constructs localized to the mCherry-Talin(FL) containing adhesions (Fig. 2*A*, quantified in Fig. 2*B*).

The localization of GFP-R11 and GFP-H50 to FAs was in good agreement with previous work (18, 30) which mapped the FA-targeting region in R11 to residues 2077–2099. However, as the GFP-VVLINAV sequence is sufficient for targeting to FAs we have further refined this FA-targeting motif in talin to just seven residues (2078–2084).

Having established that GFP-R11 fragments can localize to FAs containing Talin(FL) we next wanted to test whether deletion of R11 from Talin(FL) would reduce GFP-R11 targeting, using mini-talin constructs lacking most of the talin rod: Talin(ΔR1–R12) and Talin(ΔR1–R10) (31). Upon transfection, these mini-talins enable cell spreading of the talin null cells. GFP-R11 colocalized with Talin(FL) and Talin(ΔR1–R10) but did not colocalize with Talin(ΔR1–R12) which lacks R11–R12 (Fig. 2 *C* and *D*). The absence of colocalization was also seen for GFP-VVLINAV (*SI Appendix*, Fig. S2 *A* and *B*). Together, these data indicate that APR-containing R11 fragments localize to FAs through interaction with R11-containing talin already present at adhesion sites.

### The Canonical APR-Disrupting Gatekeeper Mutation, V2078K, Prevents R11 Self-assembly In Vitro and H50 Recruitment in Cells.

**Fig. 3.**
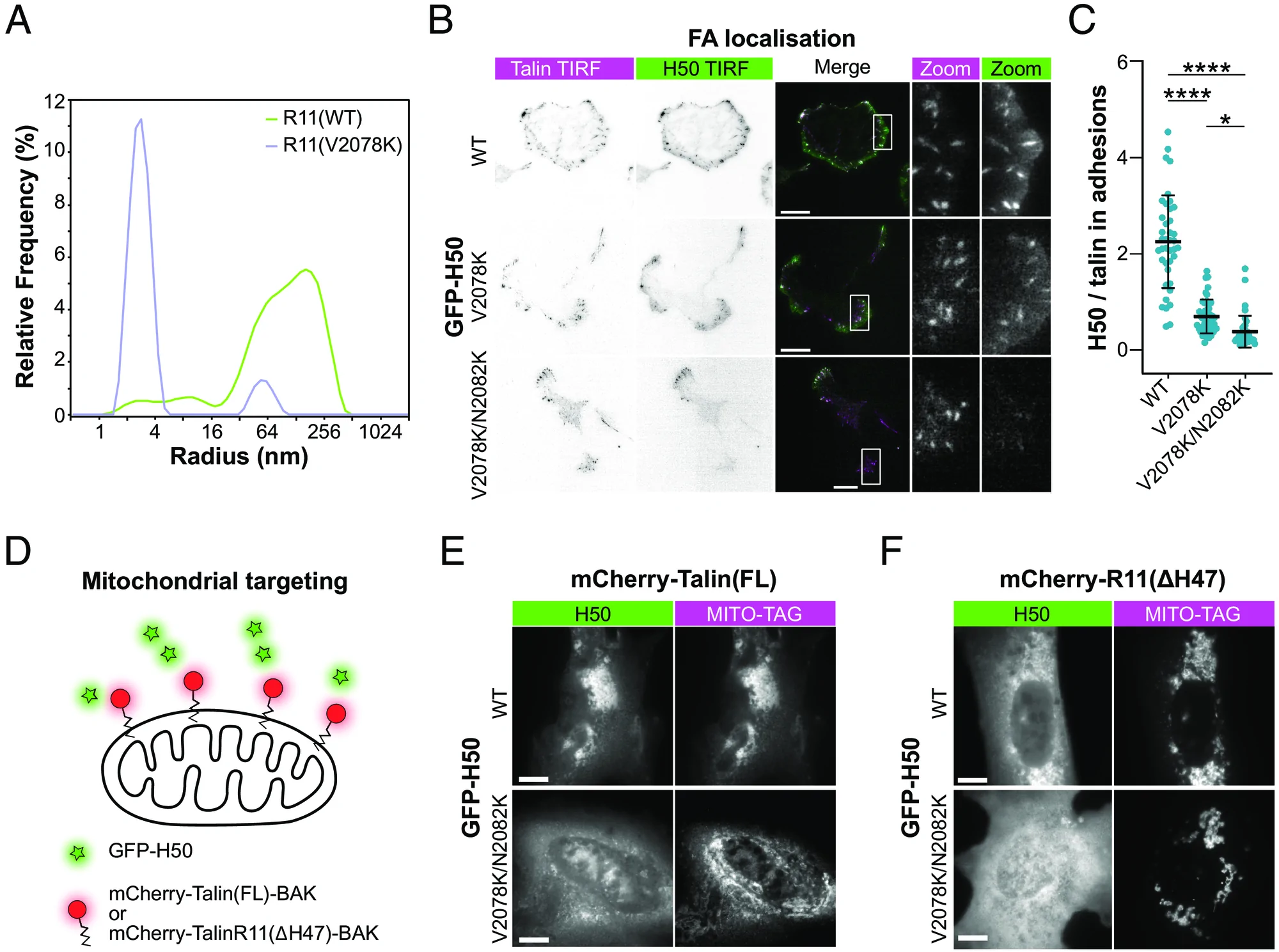
A gatekeeper mutation in the APR prevents R11 self-assembly in vitro and APR-dependent recruitment in cells. (*A*) DLS comparison of R11(WT) (green) and R11(V2078K) (blue) in the absence of reducing agent. The V2078K gatekeeper mutation significantly reduces the propensity for R11 to self-assemble. (*B* and *C*) Recruitment of GFP-H50 constructs to talin at FAs. (*B*) Representative TIRF images showing colocalization of wild-type GFP-H50 and GFP-H50 gatekeeper mutants, V2078K and V2078K/N2082K, with full-length talin. (Scale bar, 20 µm.) (*C*) Quantitative analysis of colocalization between GFP-H50 or the indicated gatekeeper mutants and full-length talin (three independent experiments, n = 35 to 40 cells). Statistical significance was assessed using Brown–Forsythe and Welch ANOVA tests. Data are presented as mean and SD from three independent biological replicates. Statistical significance is indicated as follows: *P < 0.05; **P < 0.01; ****P < 0.0001. (*D*–*F*) Recruitment of GFP-H50 to talin constructs at mitochondria. (*D*) Schematic of the mitochondrial targeting assay. mCherry-tagged Talin(FL)-BAK or a destabilized R11 construct lacking the first helix, R11ΔH47-BAK, were targeted to mitochondria and used to assess recruitment of GFP-H50 constructs independently of FAs. R11ΔH47 exposes APR1 without requiring mechanical force. (*E*) Wild-type GFP-H50, but not GFP-H50(V2078K/N2082K), is recruited to mCherry-Talin(FL)-BAK at mitochondria. (*F*) Wild-type GFP-H50, but not GFP-H50(V2078K/N2082K), is recruited to mCherry-R11ΔH47-BAK at mitochondria. (Scale bar, 10 µm.)

The APR-motif in talin R11 forms micron long fibers (Fig. 1*D*), and this self-association can be abolished by introduction of the gatekeeper point mutant, V2078K. Having validated the effectiveness of the V2078K mutation in limiting talin amyloid formation at the peptide level, we next introduced this mutation into the R11 domain. R11(V2078K) expressed and purified well, and DLS analysis confirmed that R11(V2078K) no longer forms the large species in the absence of reducing agents (Fig. 3*A*).

R11 interacts with multiple proteins and contains binding sites for tensin-3 (22) and RIAM (5) when folded, and vinculin when unfolded (23, 24). Therefore, before using this mutation for cellular analysis, we first confirmed that the V2078K mutation did not affect binding to tensin-3 or VD1 in vitro (*SI Appendix*, Fig. S3).

Having validated the efficacy of the V2078K mutant in biochemical assays, we introduced the gatekeeper mutation into our GFP-H50 construct, GFP-H50(V2078K), and studied the effect on colocalization with full-length talin at FAs (Fig. 3*B*). The mutant dramatically reduced the colocalization of GFP-H50 to FAs (Fig. 3*C*), although some FA localization was still observed. Therefore, we generated a second gatekeeper mutant, V2078K/N2082K, designed to further limit self-assembly by increasing charge repulsion within the APR. This mutant further reduced the colocalization of GFP-H50 with talin at FAs (Fig. 3 *B* and *C*).

To determine whether APR-mediated recruitment could occur independently of FAs, we used a mitochondrial targeting assay (32) in which mCherry-tagged Talin(FL) or TalinR11ΔH47 were mislocalized to mitochondria using a BAK targeting sequence, generating mCherry-Talin(FL)-BAK and mCherry-TalinR11ΔH47-BAK, respectively (Fig. 3*D*). We then examined recruitment of GFP-H50 with or without the V2078K/N2082K gatekeeper mutation. Consistent with our model, GFP-H50 was robustly recruited to both mitochondrial Talin(FL)-BAK (Fig. 3*E*) and TalinR11ΔH47-BAK (Fig. 3*F*), whereas recruitment of GFP-H50(V2078K/N2082K) was markedly reduced. These findings demonstrate that APR-mediated recruitment can occur in cells outside the FA environment and does not require integrins. Together, these data establish APR1 as a major determinant of H50 recruitment both at FAs and in an independent cellular recruitment assay.

### The V2078K Mutation in Full-Length Talin Alters Cell Morphology, Migration, Signaling, and Adhesion Dynamics.

**Fig. 4.**
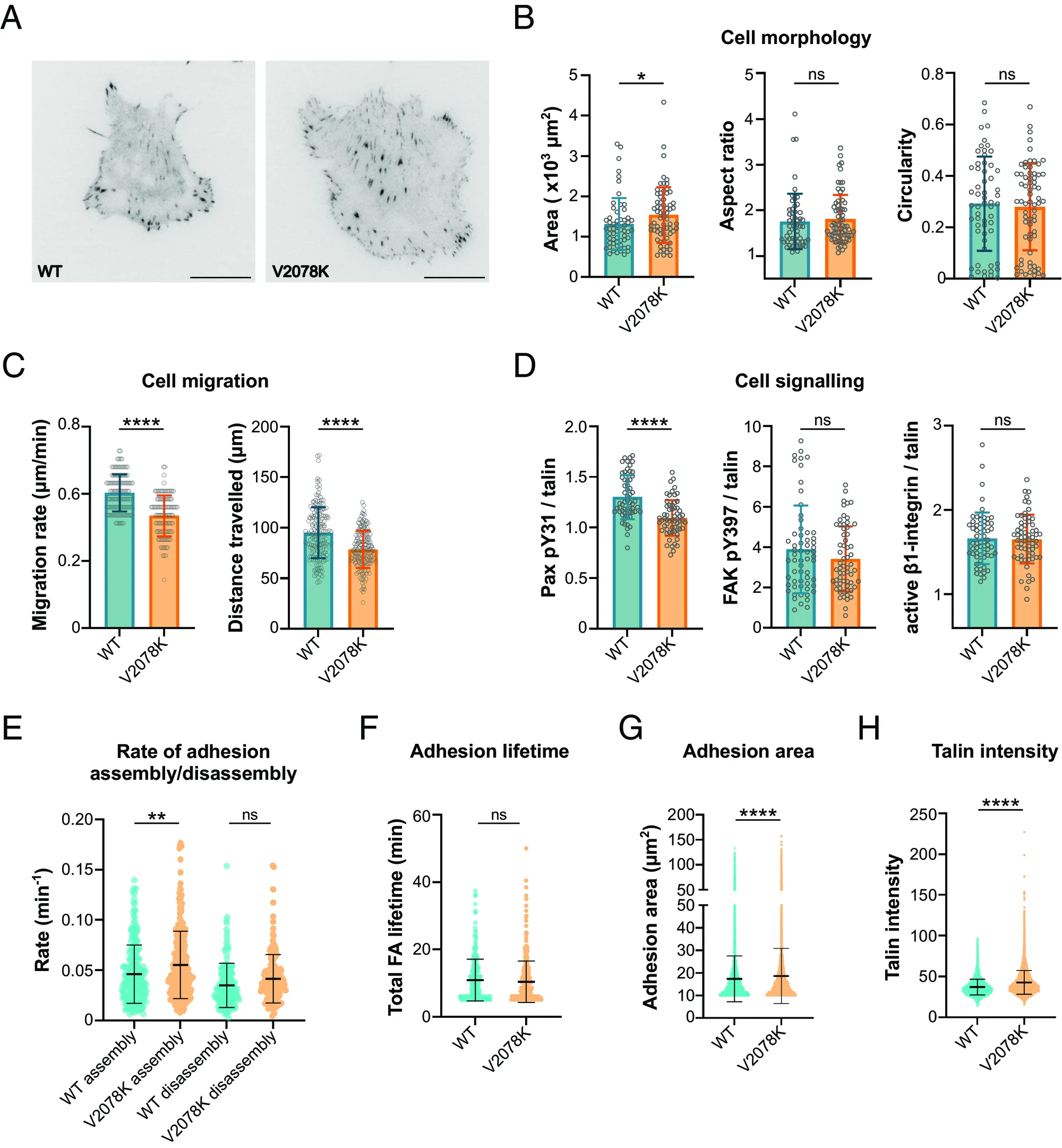
The V2078K mutation in full-length talin alters FA organization, migration, signaling, and dynamics. (*A*) Representative TIRF images of talin-null cells rescued with WT talin or talin(V2078K). Scale bars, 20 µm. (*B*) Quantification of cell morphology parameters: cell area, aspect ratio, and circularity in cells expressing WT talin or talin(V2078K) (three independent experiments, n = 67 to 76 cells per condition). Statistical significance was assessed using the Mann–Whitney *U* test. (*C*) Random migration analysis including migration rate and total distance traveled for cells expressing WT talin or talin(V2078K) (three independent experiments, n = 160 to 179 cells for migration rate and n = 175 cells for distance traveled). Statistical significance was assessed using Welch’s *t* test. (*D*) Quantification of adhesion-associated signaling markers, shown as intensity ratios of paxillin pY31, FAK pY397, and active β1-integrin relative to talin at adhesion sites (three independent experiments, n = 60 cells per condition). (*E*–*H*) FAAS analysis of FA dynamics and architecture in cells expressing WT talin or talin(V2078K). Quantification of (*E*) adhesion assembly and disassembly rates, (*F*) total FA lifetime, (*G*) adhesion area and (*H*) talin intensity. V2078K adhesions show increased assembly rate, increased adhesion area, and increased talin intensity, while disassembly rate and total FA lifetime are not significantly changed. Statistical significance was assessed as described in *Methods*. Data are presented as mean and SD unless otherwise indicated. Statistical significance is indicated as follows: ns, not significant; *P < 0.05; **P < 0.01; ****P < 0.0001.

Having validated the V2078K mutant as an effective way to reduce APR-mediated talin self-interactions, we next investigated the biological relevance of this interaction in cells. To do this, we introduced the V2078K gatekeeper mutation into full-length talin and evaluated the effects on cell morphology, migration, adhesion signaling, and adhesion dynamics. *Tln1^−/−^ Tln2^−/−^* MKF cells were transfected with either wild-type talin or talin(V2078K). Cells expressing talin(V2078K) were larger than cells expressing wild-type talin (area 1,539 ± 686.5 µm^2^ versus 1,315 ± 639.6 µm^2^), whereas aspect ratio (V2078K, 1.81 ± 0.53; WT, 1.76 ± 0.61) and circularity (V2078K, 0.28 ± 0.17; WT, 0.29 ± 0.18) were not significantly different (Fig. 4 *A* and *B*). V2078K-expressing cells showed a reduced migration rate (0.84 ± 0.15 μm/min) and reduced total distance traveled (78.43 ± 18.44 μm) relative to wild-type cells (1.01 ± 0.14 μm/min; 95.06 ± 25.33 μm, respectively) (Fig. 4*C*).

We next assessed adhesion-associated signaling by quantifying pY31 paxillin, pY397 FAK, and active β1-integrin levels at talin-positive adhesions. V2078K-expressing cells showed significantly lower pY31 paxillin relative to talin (1.09 ± 0.18) compared to wild-type cells (1.30 ± 0.22), whereas no significant differences were observed for pY397 FAK (V2078K, 3.42 ± 1.61; WT, 3.90 ± 2.17) or active β1-integrin (V2078K, 1.66 ± 0.29; WT, 1.67 ± 0.31) (Fig. 4*D*). Thus, talin(V2078K) supports integrin activation but alters adhesion-associated signaling, particularly paxillin phosphorylation.

To directly examine adhesion dynamics, we performed live-cell TIRF imaging followed by FAAS analysis (Fig. 4 *E*–*H* and *SI Appendix*, Fig. S4*A*). Talin(V2078K) adhesions showed a significantly increased assembly rate compared to wild-type adhesions, whereas the disassembly rate was not significantly changed (Fig. 4*E*). Total FA lifetime was also not significantly different between wild-type and V2078K-expressing cells (Fig. 4*F*). However, talin(V2078K) adhesions were significantly larger and contained higher talin intensity than wild-type adhesions (Fig. 4 *G* and *H*). Additional analysis following blebbistatin treatment showed that inhibition of actomyosin contractility altered the spatial distribution of adhesions and reduced the differences between wild-type and V2078K-expressing cells (*SI Appendix*, Fig. S4*B*). Together, these data indicate that disrupting APR1 does not prevent adhesion formation or integrin activation, but alters adhesion remodeling, producing larger, more talin-rich adhesions with altered assembly dynamics, reduced paxillin phosphorylation, and reduced migration in this assay.

### Talin R11 Oligomerization Is Mechanically Gated.

The finding that destabilizing R11 is sufficient to trigger self-association (*SI Appendix*, Fig. S1*F*) suggested that mechanical unfolding of the R11 domain could be a normal mechanism to mechanically regulate talin oligomerization via the APR. Our previous work on other cryptic binding sites in talin, including the multiple vinculin binding sites that are exposed upon rod domain unfolding (7, 8) and the A-kinase Anchoring Protein (AKAP) helix in R9 that binds Protein Kinase A when R9 is unfolded (33), revealed a way to assay binding to an unfolded domain at the single molecule level. When talin is put through cycles of stretching to progressively unfold each domain, followed by relaxation to allow refolding, addition of a ligand can prevent refolding of the domain to which it binds. In the subsequent stretch cycle, this is observed as the loss of the corresponding unfolding step, because that domain remains ligand-bound and unfolded.

**Fig. 5.**
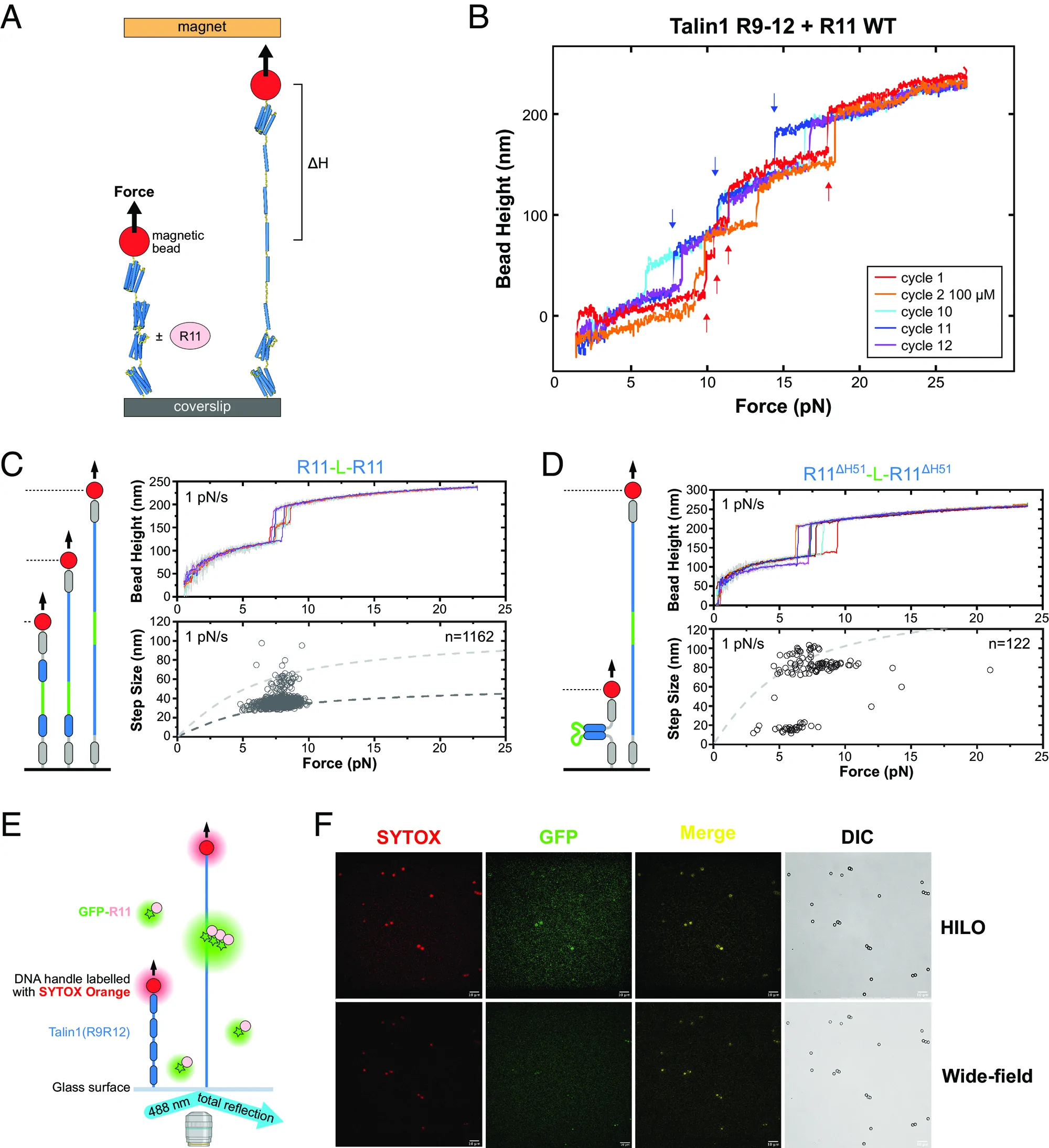
Mechanical gating of the APR in talin R11. (*A*) Schematic showing the single-molecule magnetic tweezers experiment. Talin R9–R12 is tethered between coverslip and a superparamagnetic bead using 572-base pair DNA handles. Force is exerted and domain unfolding results in stepwise jumps in bead height (∆H). (*B*) Representative consecutive force-height curves obtained from a tether of R9–R12 at a loading rate of 2 ± 0.04 pN/s are shown. The first cycle is R9–R12 alone and four unfolding steps are detected (red arrows). On cycle 2, 100 µM R11 is added and within a few cycles one unfolding step is lost (blue arrows). Once lost, this step is lost in all subsequent cycles. (*C* and *D*) Schematics showing the single-molecule detector assay experiment. Two copies of R11 [blue; either R11 (*C*) or R11ΔH51 (*D*)] are connected via an unstructured polypeptide linker (green). *Top*: Force-bead height and *Bottom*: Force-step size curves for (*C*) R11–R11 and (*D*) R11ΔH51–R11ΔH51. The R11 domains refold faithfully each time and give two discrete unfolding steps. In contrast the R11ΔH51 unfolds as a single unfolding step indicating a direct interaction between the two copies. (*E*) Schematic showing the single-molecule magnetic tweezers-TIRF experiment. Magnetic tweezers are installed over a TIRF microscope, and then the DNA handle is labeled with SYTOX Orange and can be visualized in the red channel. GFP-R11 and GFP-H50 are then added into the chamber. (*F*) Visualization of multiple beads. Upon force addition, the green GFP signal grows at the same place as the red channel signal from the SYTOX Orange indicating recruitment of soluble GFP-R11 to the stretched R9-R12 tether.

To study the talin R11-talin R11 interaction we applied single molecule magnetic tweezer approaches to mechanically stretch talin R9–R12 as described previously (7, 33) and used free talin R9–R12, R11, or H50 as the ligand (Fig. 5 *A* and *B* and *SI Appendix*, Fig. S5). In this experiment, multiple force cycles are applied to the protein. Initially, a linearly increasing force (1.5 to 30 pN at a constant loading rate of 2 pN/s) is applied to the protein tether which unfolds the domains, before the force is lowered back to ~1.5 pN to allow refolding. When stretching R9–R12 alone, four unfolding steps are observed in each cycle (red arrows in Fig. 5*B*), confirming that in the absence of other proteins, these domains exhibit switch-like behavior and unfold and refold with high fidelity. We next applied the same magnetic tweezer technique but added R9–R12, R11, or H50 as a ligand into the experiment and watched how they affected the tethered R9–R12 behavior (Fig. 5*B*). All magnetic tweezer experiments were performed in the presence of TCEP to maintain reducing conditions and minimize preformed higher-order R11 species prior to force-dependent recruitment. Strikingly, addition of R9–R12 into the chamber resulted in the loss of one unfolding step in subsequent stretch cycles (*SI Appendix*, Fig. S5), confirming that one domain was unable to refold, indicating an interaction with the tethered R9–R12 protein (*SI Appendix*, Fig. S5). A similar loss of unfolding step was seen when just R11 (Fig. 5*B*) or H50 alone (*SI Appendix*, Fig. S5) were added, enabling us to ascribe the lost unfolding step to R11. Thus, because force is applied only to the tethered R9–R12 molecule, these experiments indicate that exposure of APR1 in one mechanically stretched talin molecule is sufficient to recruit soluble R11-containing talin fragments, including R11, H50, and R9–R12, from solution.

With the single-molecule tweezer experiment we are detecting changes in the single molecule that is tethered, and so we cannot directly observe the ligand. With talin oligomerizing via the APR we next wanted to test whether; i) two molecules of R11 are sufficient to interact, and ii) one mechanically stretched R11 can recruit multiple R11 molecules from the surrounding solution. To test these scenarios, we used two variations of the single molecule experiments we had established in our lab.

### Two Molecules of R11 Are Sufficient to Interact.

Measuring binding kinetics at the single-molecule level is challenging because, when the ligand dissociates, it diffuses away, making repeated measurements difficult. We therefore used a single-molecule detector assay that we previously developed (34), in which two proteins are connected by an unstructured polypeptide ensuring that they remain in proximity after force is removed. We generated two versions of the assay: one containing two copies of R11 and a second containing two copies of R11 lacking the final helix (R11ΔH51), which destabilizes the domain (Fig. 5 *C* and *D*). The R11–R11 assay showed highly robust force-dependent unfolding and refolding of R11: the two R11 domains unfolded at around 7 pN and refolded independently over many cycles with high fidelity (>95%) (Fig. 5*C*). The absence of a cooperative rupture event in this construct indicates that, after force-induced unfolding, the two R11 domains preferentially refold independently rather than forming a stable intermolecular complex under these conditions. In contrast, the R11ΔH51–R11ΔH51 assay showed either a single large rupture step (~95 nm), or a small partial unfolding step (~15 nm) followed by a large rupture step (~80 nm), consistent with the extension change expected from release of the long linker upon complex rupture. Thus, this assay reveals that mechanical, thermal, or mutagenesis-assisted unfolding is required for R11 complexation, and establishes that exposure of APR1 is sufficient to promote direct R11–R11 interactions at the single-molecule level (Fig. 5*D*).

### One Mechanically Stretched R11 Molecule Can Recruit Multiple R11 Molecules From the Surrounding Solution.

The final single molecule experiment we performed was to combine two mature technologies, namely single-molecule magnetic tweezers and total internal reflection fluorescence (TIRF) microscopy (Fig. 5*E*). We also used highly inclined and laminated optical (HILO) microscopy. TIRF excites molecules within 100 to 200 nm of the coverslip whereas HILO excites within a few microns. In this assay the R9–R12 tether was attached to a magnetic bead via a DNA handle that was fluorescently labeled with SYTOX orange and then stretched in the absence or presence of GFP-R11 or GFP-Helix50. The tether is then observed using TIRF/HILO and Differential Interference Contrast (DIC) microscopy to visualize the bead, the tethered SYTOX Orange labeled R9–R12 molecule and the soluble GFP-R11 molecules. When the magnetic tweezer exerted around 20 pN of force on the beads, a strong GFP signal was detected colocalizing with the SYTOX Orange tethered molecule (Fig. 5*F*). This coalescence was more clearly observed using HILO, likely because the complex is forming >150 nm above the surface and is approaching the limit of the TIRF field. The use of fluorescence techniques with magnetic tweezers demonstrates that each tethered R9–R12, once mechanically unfolded, recruits soluble GFP-R11 from the solution. Although the fluorescence signal was not calibrated for molecule counting, the strong GFP signal is consistent with recruitment of more than one GFP-R11 molecule to the tether. Because force is applied only to the tethered R9–R12 molecule, recruitment of soluble GFP-R11 indicates that exposure of APR1 in one stretched talin molecule is sufficient to promote recruitment from solution.

### Vinculin Is a Mechano-Chaperone.

**Fig. 6.**
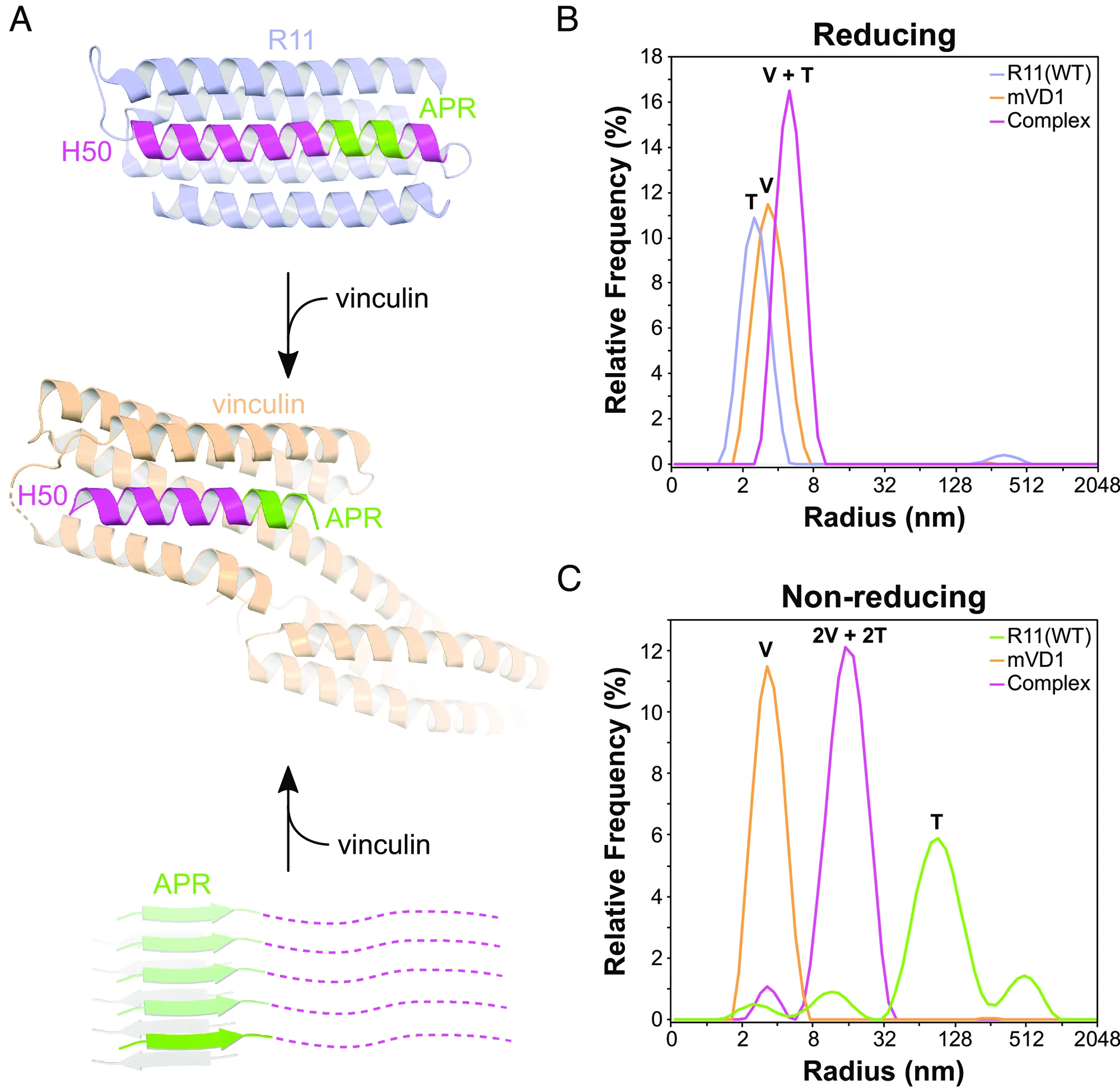
Vinculin acts as a mechano-chaperone for talin. (*A*) R11 (blue) contains a VBS (H50; magenta) in which the APR (green) is also located. Vinculin (orange) binds the exposed VBS in Helix50 and can disrupt APR-mediated talin assemblies to form a high-affinity vinculin–talin complex. R11 structure from 3dyj (19), showing residues 2074–2140. VD1 in complex with H50 from 4dj9 (24). (*B* and *C*) DLS data showing complex formation between R11(WT) and VD1. (*B*) In reducing conditions VD1 binds to H50 when R11 is in the unfolded state. Incubation of VD1 (orange) with R11 (blue) causes a complex (magenta) to form at a larger hydrodynamic radius than either VD1 or R11 alone. (*C*) VD1 can disassemble the soluble aggregates. In nonreducing conditions R11 (green) forms large species, which when mixed with VD1 (orange) results in a complex (magenta) with smaller hydrodynamic radius than R11 alone. The complex peak has a larger hydrodynamic radius than in (*B*) as it consists of an R11 dimer bound to two VD1s. See also Movie 1.

Our work defines Helix50 as a multifunctional region of talin. When R11 unfolds, this helix is a high affinity VBS (35). However, when stretched into an extended polypeptide, the VVLINAV motif creates a high-affinity APR that mediates talin self-assembly. Thus, the conformation of H50 dictates the interactions it forms: as a helix it binds vinculin, whereas as a β-strand it can bind other copies of talin. Because the APR region in H50 overlaps with the VBS, these two interactions are predicted to be mutually exclusive (Fig. 6*A*). This raised the possibility that vinculin might outcompete talin–talin interactions and thereby resolve talin assemblies.

To test this, we used DLS (Fig. 6 *B* and *C*), first in the presence of TCEP (Fig. 6*B*) where the DLS profiles showed single peaks for talin R11 (blue), vinculin D1 (orange), and the resultant 1:1 complex (magenta) that forms when they are mixed. In contrast, in the absence of reducing agent (Fig. 6*C*) talin R11 alone forms the big species (as seen in Fig. 1*B*), but this big species is completely lost when mixed with vinculin D1 (magenta). We note here the increased size of the R11–VD1 complex relative to the equivalent peak in Fig. 6*B*, which is due to R11 being a disulfide-linked dimer and bound to two VD1 molecules. However, the DLS unequivocally shows that vinculin has a previously unrecognized chaperone ability for talin as it can resolve these large species.

## Discussion

A major challenge in studying potential integrin-binding proteins in cells is that recruitment to IACs can occur either through direct integrin binding, as is the case for proteins such as kindlin and talin, or indirectly through interactions with adhesion-associated scaffolds. Here we report that talin R11 contains a high-affinity self-interaction site that mediates recruitment of R11-containing fragments to IACs through interactions with talin already present at adhesion sites. Deletion of R11 from talin at FAs abolishes FA-targeting of GFP-R11 constructs (Fig. 2), indicating that recruitment is not mediated by integrins. Importantly, mitochondrial-targeting experiments show that APR1-mediated recruitment can also occur in cells outside FAs, demonstrating that the interaction does not require integrins or the FA environment.

We present data showing functional aggregation of talin, mediated by a cryptic APR, buried within the folded R11 bundle. The APR region in R11 was previously mapped as the second integrin-binding site (IBS2), and its localization to IACs was concluded to arise from direct binding to integrin beta cytoplasmic tails. Our data support a primary function in talin self-interaction and allow the previous integrin-binding observations to be reinterpreted as talin oligomerization. Therefore, we propose that this cryptic VVLINAV talin-binding motif in R11 be renamed from IBS2 to APR1, because the historical IBS2 designation implies direct integrin binding and no longer accurately reflects the function of this motif.

### A Context-Dependent Binary Switch.

The talin rod domains serve as force-dependent binary switch domains, as tension can convert them from folded “0” states to unfolded “1” states. This process is reversible when tension is released and each state has different functions. These binary switches form a central part of the IACs in most cells. Strikingly, when the R11 helical bundle unfolds, it reveals Helix50, which itself has multiple functions. If vinculin is present, Helix50 can mediate a high affinity connection to the actin cytoskeleton. However, force applied to exposed α-helices can unravel them and promote conversion to β-strand conformations (36). The forces required to unfold R11 in our single-molecule experiments are within the range experienced by talin rod domains in mechanically loaded FAs, and unfolding of an exposed helix can occur at very low forces. In the context of talin, if vinculin is not available, tension on Helix50 is therefore expected to favor extension of the helix and formation of intermolecular β-sheet interactions, stabilizing the high-affinity talin-binding site. At the single-molecule level, we show that mechanical exposure of APR1 in a stretched talin molecule enhances recruitment of R11-containing molecules from solution and promotes intermolecular assembly (Fig. 5). This is still a binary switch domain, as both ligands for the unfolded R11 state (vinculin and talin) require the R11 domain to be unfolded. The discovery of a cryptic talin-binding site that is exposed when R11 is in the unfolded “1” state and unbound to vinculin indicates a context dependence to what the switch engages and further expands the complexity and sophistication of mechanical signaling at adhesion sites. This interdependence between force and ligand availability represents another example of an “AND/OR” logic gate in talin and further expansion of a talin code (2). The observation that vinculin D1 can disassemble preexisting APR-mediated assemblies demonstrates direct competition between these two binding modes and supports the concept that mechanically exposed R11 can adopt alternative signaling states depending on ligand availability and local molecular context. Talin is a synaptic scaffold in the brain and coordinates signaling via its binary switch domains (9, 10, 37) so understanding the roles of each switch and the logic underpinning their functioning is crucial to understand mechanical signaling and computation in the brain.

Isolated R11/H50 fragments depend on APR-mediated self-association for recruitment, whereas full-length talin(V2078K) retains the talin head, integrin-binding activity, and other adhesion-targeting mechanisms required for adhesion localization. The cellular consequences of disrupting APR1 suggest that its primary role may not be to initiate adhesion assembly but rather to regulate the behavior of mature adhesions. Although V2078K talin supports integrin activation and FA formation, the resulting adhesions are larger, more talin-rich, and exhibit altered assembly dynamics and signaling properties. These findings are consistent with a model in which force-dependent exposure of APR1 provides an additional mechanism for adhesions to sense and respond to mechanical load. Loss of this response does not prevent adhesion formation but instead alters adhesion remodeling and is associated with reduced migration. Thus, APR1 appears to contribute to mechanical regulation within adhesions rather than functioning solely as a recruitment module.

### Harnessing Protein Unfolding and Aggregation in Mechanotransduction.

The role of protein unfolding in mechanotransduction is now firmly established (38, 39). Integrin, force exerted on talin, and vinculin have all been shown to “trigger” conformational changes in talin (7, 40–42). However, as mechanotransduction involves unfolding of talin rod domains it raises an intriguing question: what happens to the exposed hydrophobic residues normally resident in the domain core? In talin (7) and vinculin (43) force-dependent domain unfolding/refolding happens with high fidelity which is no mean feat, but with only 13 of the 61 helices in the talin rod having known function (either VBS or AKAP helices) when exposed, this leaves 48 amphipathic helices exposed and potentially unfolded, which indicates that mechanotransduction will lead to a lot of exposed hydrophobic residues.

Often protein unfolding is harmful, as exposure of hydrophobic residues can result in nonspecific aggregation, yet somehow the mechanotransduction machinery seems to circumvent this issue. Cells protect themselves from such aggregates, which are well known drivers of disease, by using chaperones that dissolve and refold these aggregates or direct them for destruction (44).

### Vinculin as a Mechano-Chaperone.

As unwanted protein aggregation is undesirable for healthy function, APR sequences are actively protected from aggregation in the cell. This can be by forming part of a protein-interaction surface, or by being buried in the hydrophobic core of proteins. Alternatively, they can be protected by naturally occurring gatekeeper residues (45) that prevent aggregation should these regions become exposed.

In talin these motifs are not protected by gatekeeper residues, but are cryptic, indicating that mechanical exposure of these APR motifs might facilitate functional aggregation. We propose that aggregation of talin, which occurs in response to mechanotransduction-induced unfolding, is harnessed to build adhesion complexes by forming self-interactions, generating an “intracellular meshwork” and in this work we directly link these self-interactions to mechanical forces and the redox environment. Importantly, resolving these aggregates typically requires interaction with specific chaperones (44), and we show that vinculin is able to resolve talin aggregation, identifying a novel “mechano-chaperone” function for vinculin (Fig. 6).

### Potential Redox Sensitivity of APR1.

Last, our in vitro data show that exposure of Cys1978 can stabilize higher-order R11 species under nonreducing conditions, suggesting a potential link between mechanical unfolding and redox sensitivity. We have not directly measured redox regulation of APR1 in cells, so we consider it an interesting possibility for future work that more oxidizing conditions could stabilize mechanically exposed R11 and strengthen talin–talin interactions. Notably, 12 of the 13 talin switch domains contain buried cysteines, suggesting that interplay between force-dependent unfolding and cysteine exposure may be a broader feature of talin mechanobiology.

In summary, we present here a new feature of mechanotransduction, mediated by mechanically gated APRs in talin switches. Identification of a gated talin-binding site in talin reveals a new function of talin switches and a potential new link between mechanical- and redox-signaling.

## Methods

Full details can be found in the *SI Appendix*, *Supplementary Methods*.

### Biochemistry.

#### Peptides, plasmids, and cloning.

All talin constructs were based on the mouse talin-1 sequence (UniProt ID: P26039). Synthetic peptides of mouse talin-1 VVLINAV (2078–2084) and gatekeeper mutant, KVLINAV were ordered from GLBiochem (Shanghai).

#### Protein expression and purification.

Talin-1 R11 constructs were expressed in BL21(DE3) and purified by nickel-affinity and anion exchange chromatography. Pure proteins were dialyzed against PBS pH 7.4.

#### AFM.

The VVLINAV and KVLINAV peptides were dissolved in Milli-Q water and allowed to self-assemble at room temperature. The samples were deposited onto freshly cleaved mica surfaces (Agar Scientific, F7013) and incubated for 10 min. Fibrils were imaged using a Multimode 8 AFM with a Nanoscope V (Bruker) controller operating under peak-force tapping in the ScanAsyst mode. Height channel images were collected with 2048 × 2048 pixels each.

#### DLS.

Protein samples were prepared at 50 µM in PBS pH 7.4 in either the presence or absence of 0.5 mM TCEP and loaded into nanoDSF grade standard capillaries (PR-C002; NanoTemper). DLS data were collected using a Prometheus Panta (NanoTemper), performing 10 high sensitivity scans of each capillary at 25 °C.

#### Fluorescence Polarization (FP).

Assays were performed in triplicate with 500 nM fluorescein-labeled peptide and a 2-fold serial dilution of protein in PBS pH 7.4, 0.01% v/v Tween-20, 0.5 mM TCEP. FP was measured using a Hidex Sense plate reader (Hidex) at 25°C (excitation: 485 ± 10 nm; emission: 535 ± 20 nm). Data were analyzed using OriginPro (OriginLab Corporation) and K_d_ values were calculated using a single site with nonspecific binding equation.

#### Cell culture.

*Tln1^−/−^Tln2^−/−^* mouse kidney fibroblast (MKF) cells (3) and NIH3T3 fibroblasts were used and maintained under humidified conditions at 37 °C and 5% CO_2._ High glucose Dulbecco’s modified Eagle medium (DMEM) supplemented with 10% fetal bovine serum (FBS) was used. The cells were routinely tested for *Mycoplasma* contamination.

#### Transfection and expression constructs.

Cells were transfected with 1 µg/ 2 kb of plasmid DNA per 10^6^ cells by electroporation using Neon NxT transfection system (Thermo Fisher Scientific). The electroporation parameters were 1,350 V, 30 ms, one pulse per 10^6^ cells. Transfected cells were incubated overnight to allow recovery posttransfection. Talin expression constructs are detailed in the *SI Appendix*, *Supplementary Methods*.

#### Migration and wound closure assay.

Artificial wounds were created using 10 μL pipette tips and washed with PBS to remove dead and detached cells. Wound healing was monitored by time-lapse imaging for 24 h at 10 min intervals using a Nikon Eclipse Ti2 Fluorescence Microscope. Migration rate and distance traveled were measured in ImageJ (Fiji).

#### Immunostaining.

Fixed cells were immunostained for phosphorylated paxillin (pY31 paxillin), phosphorylated FA kinase (pY397 FAK) and activated β1-integrin antibodies, followed by fluorescently labeled secondary antibodies according to the standard immunostaining protocol. Cells were imaged with a TIRF microscope (Evident Olympus IX83) using a 100× oil objective. Images were captured from both epifluorescence (EPI) and TIRF configurations.

#### Image analysis.

For adhesion segmentation we used the WEKA trainable segmentation plugin (46) and classifier to detect FAs (47) in ImageJ. All adhesions above the size of 5 pixels were selected and quantified the fluorescence intensity to obtain the ratio between talin and protein of interest within adhesion sites. Adhesion distance from the cell edge was determined by first selecting the cell outline in ImageJ. A Euclidean Distance Map (EDM) was then generated within the cell interior using the Distance Map function in ImageJ (22). The mean intensity of adhesions mapped onto the EDM was used to quantify their relative position within the cell.

#### Live cell imaging and adhesion dynamics analysis.

Time-lapse imaging was performed for 1 h at 30 s intervals with TIRF microscope (Evident Olympus IX83) using 100× oil objective. Fluorescence signals were obtained from excitation wavelength 561 nm (mScarlet). Images were captured from TIRF configuration. Laser power and exposure time were adjusted to reduce photobleaching and maintain optimal signal to noise ratio. Time lapse images were submitted to the focal adhesion analysis server (FAAS) (48).

#### Mitochondria recruitment experiments.

NIH3T3 fibroblasts were cotransfected with the indicated GFP-tagged Talin polypeptide and mCherry-tagged mitochondria targeting constructs (generated by fusing the C-terminal portion of BAK to the C-terminus of the construct of interest) as described previously (32). Transfection was performed using Lipofectamine 3000 (Invitrogen) using equal amounts of GFP-tagged and mCherry-tagged plasmids, according to the manufacturers’ instructions. Images of the GFP and mCherry constructs were acquired using a Zeiss Axio Observer.Z1 Widefield microscope equipped with a Zeiss Plan-Apochromat 63×/1.40 Oil objective. Samples were illuminated with a Colibri 7 LED system (475 module for GFP and 567 module for mCherry) and a quad emission filter (410 to 440, 499 to 529, 579 to 604, 659 to 759). Images were collected with a Zeiss AxioCam 506 camera.

#### Single-molecule manipulation.

Talin R9–R12 constructs were immobilized on glass coverslips in a laminar flow chamber via HaloTag/Halo-ligand chemistry and tethered to 3 µm paramagnetic beads through biotin–streptavidin interactions. To prevent flow-induced unfolding of the R9–R12 construct during solution exchange, a buffer-isolation membrane well array was employed (49). The experiments were carried out at a fixed loading rate of 2 pN/s.

#### TIRF-magnetic tweezer single molecule fluorescence experiment.

A 3-kbp DNA handle was introduced to act as a spacer between the magnetic bead and the surface. The synthesis of the DNA handle was carried out as described previously (50). Fluorescence images were acquired at 0° (normal) under widefield illumination and at 56 to 58° incidence angle under Highly inclined and Laminated Optical sheet (HILO) illumination, with either 488 nm laser (for GFP) or 561 nm laser (for SYTOX orange), using a 100× Apo TIRF objective lens with NA1.49. Image processing was performed using ImageJ.

## Supplementary Material

Appendix 01 (PDF)

Movie S1. **Animated version of Figure 6A showing how vinculin acts as a mechano-chaperone for talin.** When R11 is folded, H50 and the APR are cryptic inside R11 which is able to bind to “0” state binders like tensin-3. Force on talin leads to unfolding of R11 which exposes H50 which can either bind to vinculin, or be extended to a beta-sheet form where it presents the high affinity Aggregation-Prone Region 1 (APR1) which can bind to other talin molecules. Vinculin can resolve these talin multimers by binding to the VBS in H50 demonstrating a novel role of vinculin as a mechano-chaperone for talin.

## Data Availability

All study data are included in the article and/or supporting information.
