# Supplementary material for: An aggregation-prone region in talin controls talin self-interactions and regulates integrin adhesion complex dynamics
Source: Proc Natl Acad Sci U S A. 2026 Nov 4;123(45):e2602326123. doi: 10.1073/pnas.2602326123 (PMC13752342; doi:10.1073/pnas.2602326123)
Supplement: Supplementary file 1 [file pnas.2602326123.sapp.pdf]

Supplementary information for

An aggregation-prone region in talin controls talin self-interactions and regulates integrin adhesion complex dynamics

Neil J. Ball[1], Paula Turkki[2,3], Yanyu Guo[4,5], Mahmudul H. Tanvir[2], Shimin Le[4,5], Jasmine Fei Li Chin[5], Karen B. Baker[6], Jingxing Luo[1], Paul Atherton[7], Nicholas H. Brown[8], Vesa P. Hytönen[2,3], Jie Yan[4,5] and Benjamin T. Goult[1,9,*]

1 Department of Biochemistry, Cell & Systems Biology, Institute of Systems, Molecular & Integrative Biology, University of Liverpool, Crown Street, Liverpool L69 7ZB, U.K.
2 Faculty of Medicine and Health Technology, Tampere University, 33520 Tampere, Finland
3 Fimlab Laboratories, 33520 Tampere, Finland
4 Department of Physics, National University of Singapore, Singapore 117542
5 Mechanobiology Institute, National University of Singapore, Singapore 117411
6 School of Biosciences, University of Kent, Canterbury, Kent, CT2 7NJ, UK
7 Department of Molecular and Clinical Cancer Medicine, Institute of Systems, Molecular and Integrative Biology, University of Liverpool, Liverpool L69 3GE, UK.
8 Department of Physiology, Development and Neuroscience, University of Cambridge, Downing St., Cambridge CB2 1DY, UK.
9 Liverpool Interdisciplinary Neuroscience Centre, University of Liverpool, Liverpool, L9 7AL, U.K.

* To whom correspondence may be addressed. Email: b.t.goult@liverpool.ac.uk

Supplementary Methods

Peptides, plasmids and cloning

The R11(V2078K) and R11(C1978A)-Cys constructs were ordered as synthetic genes in pET151d-TOPO (GeneArt; Life technologies). R11(ΔH47) was subcloned into the XmaI/SacI sites of pET47b using ligation-independent cloning (LIC). GFP-R11 was subcloned into the XmaI/SacI sites of a modified pET47b (eGFP(A206K) inserted between the His-Tag and HRV-3C sites) using LIC. R11(H50) gatekeeper constructs (V2078K, V2078K/V2079K and V2078K/N2082K) were ordered as synthetic genes (GeneArt; Life Technologies) and subcloned into a modified eGFP-C1 vector (MCS replaced with a LIC compatible MCS) using LIC. The mCherry-R11(ΔH47)-BAK mitochondrial targeting construct was ordered as a synthetic gene (GeneArt; Life Technologies) in pcDNA3.1(hygro+).

Whole plasmid sequencing was performed by Plasmidsaurus using Oxford Nanopore Technology with custom analysis and annotation to verify all constructs. The constructs generated for this study have been deposited in Addgene (https://www.addgene.org/Ben_Goult/).

Biochemistry

Protein expression and purification

R11 and GFP-R11 constructs were expressed in BL21(DE3). Cells were grown in LB supplemented with 100 µg/mL ampicillin at 37°C. Protein expression was induced with 0.5 mM IPTG at OD_{600} ~0.6 and cells were incubated overnight at 20°C. Cells were harvested by centrifugation, resuspended in lysis buffer (50 mM Tris pH 8, 250 mM NaCl, 5% v/v glycerol) and stored at -20°C.

Proteins were purified using Nickel-affinity, followed by anion-exchange chromatography. Briefly, cells were thawed and supplemented with 1 mM PMSF, 1 mM TCEP and 0.2% v/v Triton X-100. Cells were lysed by sonication and cell debris removed by centrifugation. The supernatant was applied to a 5 mL HisTrap FF (Cytiva) using an Akta Start (Cytiva). The column was washed with 15 column volumes (CV) of wash buffer (50 mM Tris pH 8, 600 mM NaCl, 30 mM imidazole, 5 mM $MgCl_2$, 5 mM ATP, 5% v/v glycerol, 0.2% v/v Triton X-100) followed by 15 CV of nickel buffer A (20 mM Tris pH 8, 250 mM NaCl). Bound protein was eluted across a 14 CV linear imidazole gradient (0 to 300 mM) followed by 4 CV at 500 mM. Fractions containing the protein of interest were pooled and either diluted with 4.5 volumes 20 mM Tris pH 8 or dialysed against 20 mM Tris pH 8, 50 mM NaCl overnight and applied to a 5 mL HiTrap Q HP (Cytiva). Bound protein was eluted across a 14 CV linear NaCl gradient (0 to 600 mM) followed by 4 CV at 1 M. Fractions containing the protein of interest were pooled and dialysed against PBS pH 7.4 overnight. Protein was concentrated, flash-frozen in liquid nitrogen and stored at -80°C.

Fluorescence Polarisation (FP)

Peptides were synthesised by GLBiochem (Shanghai) with either an N-terminal or C-terminal non-native cysteine. These included;
Talin-1 H50 WT (C-EDPETQVVLINAVKDVAKALGDLISATKAAAG),
Talin-1 H50 V2078K (C-EDPETQKVLINAVKDVAKALGDLISATKAAAG),
Talin-1 H50 V2078K/V2079K (C-EDPETQKKLINAVKDVAKALGDLISATKAAAG),
Talin-1 H50 V2078K/N2082K (C-EDPETQKVLIKAVKDVAKALGDLISATKAAAG) and TNS3(692-712) (LDIDQSIEQLNRLILELDPT-C).
Maleimide-fluorescein dye (Thermo Fisher Scientific) was coupled to the peptide following the manufacturer's protocol.

Cell Biology

Talin expression constructs

Full-length constructs
Talin 1–2541, C-terminal mCherry / mScarlet
Talin 1–2541 (V2078K), C-terminal mScarlet
Mini-talin constructs
ΔR1–10: 1–490 + 1974–2541, C-terminal mCherry
ΔR1–12: 1–490 + 2296–2541, C-terminal mCherry
Domain fragments
R11: 1974–2140, N-terminal mEGFP
VVLINAV: 2078–2084, N-terminal mEGFP
Helix-50: 2072–2104, N-terminal mEGFP
Helix-50 mutants: N-terminal EGFP V2078K; V2078K/N2082K

The mini-talin constructs were previously described (Rahikainen et al., 2019) and the domain fragments were cloned as described in the Peptides, plasmids and cloning section of these methods.

Migration and wound closure assay

Cells were trypsinised and seeded onto 12-well plates coated with 15 µg/mL fibronectin and incubated for 90 min to allow the transfected cells to attach. Following incubation, the medium was replaced with fresh DMEM+10% FBS to remove non-transfected and non-adherent cells. Cell migration rate was measured by ImageJ (Fiji) using the MTrackJ plugin (56, 57).

For wound closure assays, transfected cells were seeded on 24-well plates coated with 15 µg/mL fibronectin and incubated for 48 h to reach the maximum cell confluency. Distance travelled was measured using ImageJ.

Immunostaining and imaging

Transfected cells were seeded on 15 µg/mL fibronectin coated, 8-well glass-bottom µ-slides (ibidi) and incubated for 24 h. Following incubation, cells were fixed with 4% paraformaldehyde. Fluorescence signals were obtained from excitation wavelength

488 nm (GFP, Alexa Fluor 488) and 561 nm (mCherry, mScarlet). Laser power and exposure time were adjusted to reduce photobleaching and maintain optimal signal to noise ratio.

Live cell imaging and adhesion dynamics analysis

WT and V2078K Tln1 transfected cells were seeded on 15 µg/ml fibronectin coated 8-well glass-bottom µ-slide (ibidi) and incubated overnight. Following incubation, the culture medium was replaced with 10% FBS containing FluoroBrite™ DMEM (Thermo Fisher Scientific). Time-lapse imaging was performed for 1 h at 30 s intervals using a Total Internal Reflection Fluorescence (TIRF) microscope (Evident Olympus IX83) with a 100x oil objective. Fluorescence signals were acquired using 561 nm excitation to detect mScarlet-labelled talin. Images were acquired in TIRF mode. Laser power and exposure time were adjusted to minimise photobleaching and maintain optimal signal-to-noise ratio.

Time-lapse images were submitted to the Focal Adhesion Analysis Server (FAAS) (53). The detection threshold and minimum adhesion size were set to 2 and 10 respectively, with all other parameters kept at default values. FAAS output was used to quantify adhesion assembly and disassembly rates, the duration of assembly, disassembly and stable phases, total phase length, adhesion area, axial ratio, adhesion signal intensity and focal adhesion alignment index.

Blebbistatin treatment

WT and V2078K Tln1 transfected cells were seeded on 15 µg/ml fibronectin coated 8 well glass bottom µ-slide (ibidi) and incubated overnight. Following incubation, the cells were treated with 25 µM blebbistatin (in DMSO) for 3 h. After that, the cells were fixed with 4% PFA and imaged with TIRF microscope as described previously.

Single-Molecule Manipulation

Instrument and calibration

These experiments were performed as previously described (7), using a custom-built high-force magnetic tweezers instrument capable of applying forces up to 100 pN with an extension resolution of approximately 1 nm (58, 59). The force-calibration was based on a standard calibration curve (6). In experiments, the curve requires a single reference point of force versus magnet-bead distance data. The experiments used the well-known DNA overstretching transition (7) as the reference point. The resulting overall uncertainty of force-calibration is less than 5%.

Single-molecule detector construction

The recombinant protein constructs were designed to consist of two R11 domain repeats (or the mutant R11ΔH51) connected by a long, flexible linker sequence. This insert was cloned into a modified pET151 plasmid (6His-AviTag-2xI27-Intein-2xI27-SpyTag) using seamless ligation. All constructs were verified by Sanger sequencing.

The final sequence of R11-L-R11 (coloured as in Fig. 5C,D) is 6His-Avi-2XI27-R11-Long Linker-R11-2XI27-SpyTag:

HHHHHHGKPIPNPLLGLDSTENLYFQGIDPFTGLNDIFEAQKIEWHEGGGSGLIEVE
KPLYGVEVFVGETAHFEIELSEPDVHGQWKLKGQPLAASPDAEIIEDGKKHILILHNA
QLGMTGEVSFQAANTKSAANLKVKELGGGSGLIEVEKPLYGVEVFVGETAHFEIELS
EPDVHGQWKLKGQPLAASPDAEIIEDGKKHILILHNAQLGMTGEVSFQAANTKSAA
NLKVKELGGGSGKLGNRGTQACITAASAVSGIIADLDTTIMFATAGTLNREGAETFAD
HREGILKTAKVLVEDTKVLVQNAAGSQEKLAQAAQSSVATITRLADVVKLGAASLGA
EDPETQVVLINAVKDVAKALGDLISATKAAAGKVGDDPAVWQLKNSAKVMVTNVTSL
LKTVKAVEDEATGGGSGEFPPAPPLPGDSGTIIPPPPAPGDSTTPPPPPPPPPPPPP
LPGGVCISSPPSLPGGTAISPPPPLSGDATIPPPPPLPEGVGIPSPSSLPGGTAIPPPP
PLPGSARIPPPPPPLPGSAGIPPPPPPLPGEAGMPPPPPPLPGGPGIPPPPPFPGG
PGIPPPPPGMGMPPPPPFGFGVPAAPVLPGSGGGSGGNRGTQACITAASAVSGIIA
DLDTTIMFATAGTLNREGAETFADHREGILKTAKVLVEDTKVLVQNAAGSQEKLAQA
AQSSVATITRLADVVKLGAASLGAEDPETQVVLINAVKDVAKALGDLISATKAAAGKV
GDDPAVWQLKNSAKVMVTNVTSLLKTVKAVEDEATLEGGGSGLIEVEKPLYGVEVF
VGETAHFEIELSEPDVHGQWKLKGQPLAASPDAEIIEDGKKHILILHNAQLGMTGEV
SFQAANTKSAANLKVKELGGGSGLIEVEKPLYGVEVFVGETAHFEIELSEPDVHGQ
WKLKGQPLAASPDAEIIEDGKKHILILHNAQLGMTGEVSFQAANTKSAANLKVKELG
GGSGAHIVMVDAYKPTK

The H51 sequence is underlined and this is deleted in the R11$^{\Delta 51}$-L-R11$^{\Delta 51}$ construct. The long flexible linker (182 aa) is from FH1 and a GGGSG short linker was inserted between each component. Some additional residues at the seams are the enzyme digestion sites.

The confirmed plasmids were co-transformed with a secondary plasmid encoding the biotin ligase BirA into *E.coli* BL21(DE3) competent cells. Protein expression was induced in the presence of D-biotin to enable site-specific biotinylation of the AviTag by BirA. Following cell lysis, the 6xHis-tagged fusion proteins were purified using immobilised metal affinity chromatography. The final protein constructs were buffer-exchanged and stored at -80°C.

TIRF-Magnetic tweezer single molecule fluorescence experiment

Imaging was performed on the iLAS2 total internal reflection fluorescence (TIRF) system, based on a Nikon Eclipse Ti-2 inverted microscope (Nikon Instruments) equipped with a motorised TIRF illuminator (GATACA systems), a scientific CMOS camera (ORCA-Fusion BT, Hamamatsu), controlled by MetaMorph software (Molecular Devices) and Modular V2.0 GATACA software. We integrated the iLAS2_TIRF system with a pre-calibrated magnet, controlled by an MP-285 step motor to apply force to M270 magnetic beads. The channel treatment process and the working buffer remained consistent with the protocol described previously.

The DNA handle was covalently attached to the M270 beads (Dynabeads M270-epoxy) via a thiol-epoxy reaction with the epoxy surface. The opposite end of the DNA handle was linked to the Talin R9-R12 AVI-tag via streptavidin. 10 μM GFP-H50 (PBS pH 7.4, 1 mM TCEP) was pre-incubated with talin R9-R12 overnight at room temperature. To facilitate the visualisation of the talin R9-R12 location under the bead, 50 nM SYTOX Orange DNA dye (Thermo Fisher Scientific, S11368) was introduced during the experiment to label the DNA handle.

Supplementary Figures

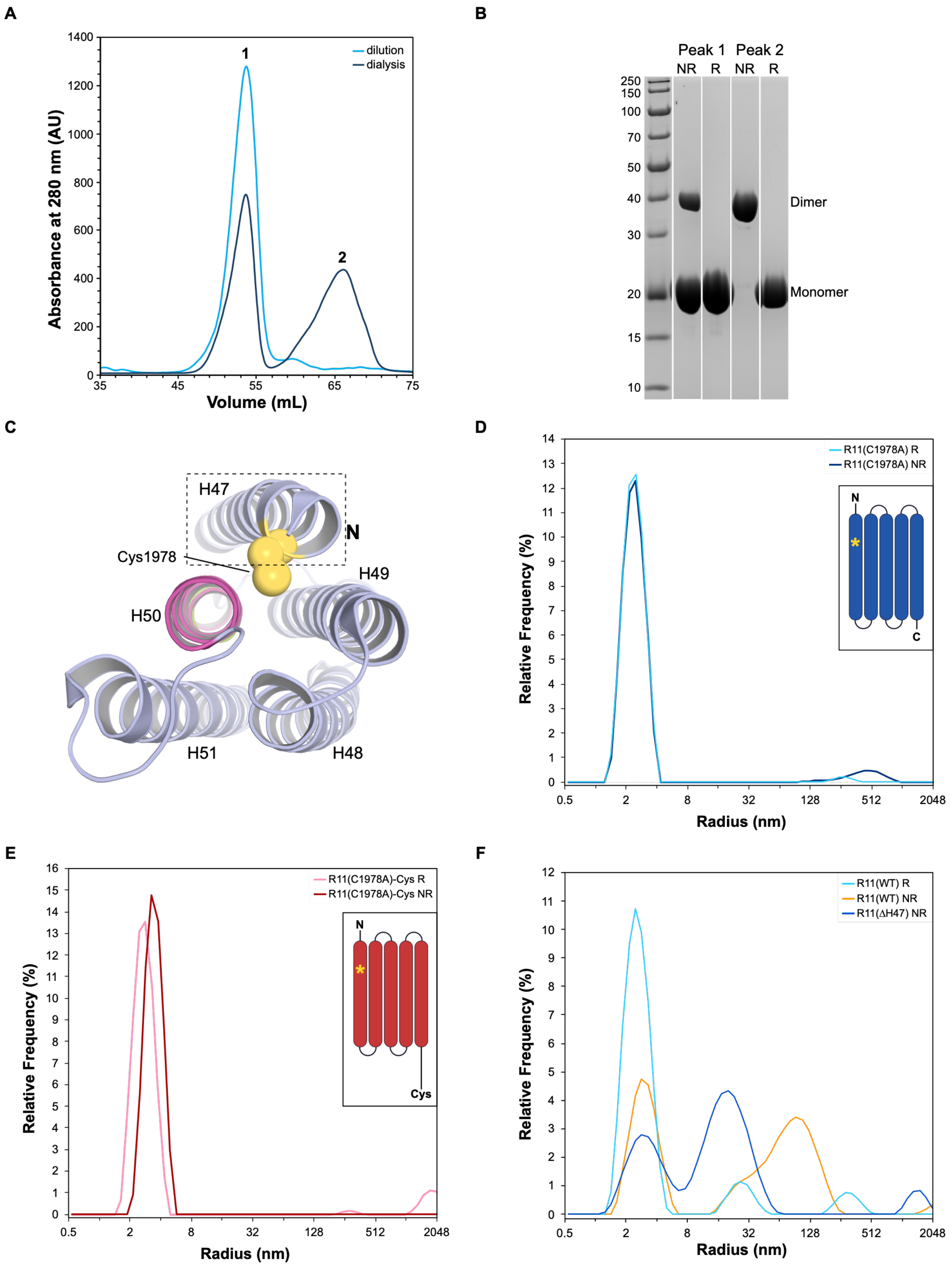

Supplementary Figure 1. **Talin R11 can form big species in solution. (A)** Two anion exchange chromatography traces of R11. When dialysed overnight, the R11 eluted as two distinct peaks (dark blue). Omitting the dialysis stage resulted in a single peak (cyan). **(B)** Analysis of the anion exchange peaks by non-reducing (NR) and reducing (R) SDS-PAGE highlights that peak 1 contains a mixture of monomeric and disulphide crosslinked R11, whereas peak 2 is solely

composed of disulphide-linked R11. **(C)** Talin R11 contains a single buried cysteine residue, Cys1978 in the first helix (H47). **(D-E)** The C1978A mutant of R11 does not form large species and dimerisation itself is not sufficient. DLS intensity distribution of **(D)** R11(C1978A) and **(E)** R11(C1978A)-Cys. The R11(C1978A) mutation does not form higher-order species in either reducing (light blue) or non-reducing (dark blue) conditions. In contrast, R11(C1978A) with a cysteine placed after a short linker at the C-terminus of R11 (schematic inset) can dimerise but does not form higher-order species in either reducing (pink) or non-reducing (dark red) conditions. This demonstrates that the higher-order species seen with R11(WT) is significantly larger than a dimer and that the formation of a disulphide-linked dimer alone is insufficient for formation of a high-order species. **(F)** R11(ΔH47), which lacks the first helix containing the cysteine, forms both monomer and higher-order species even in non-reducing buffer.

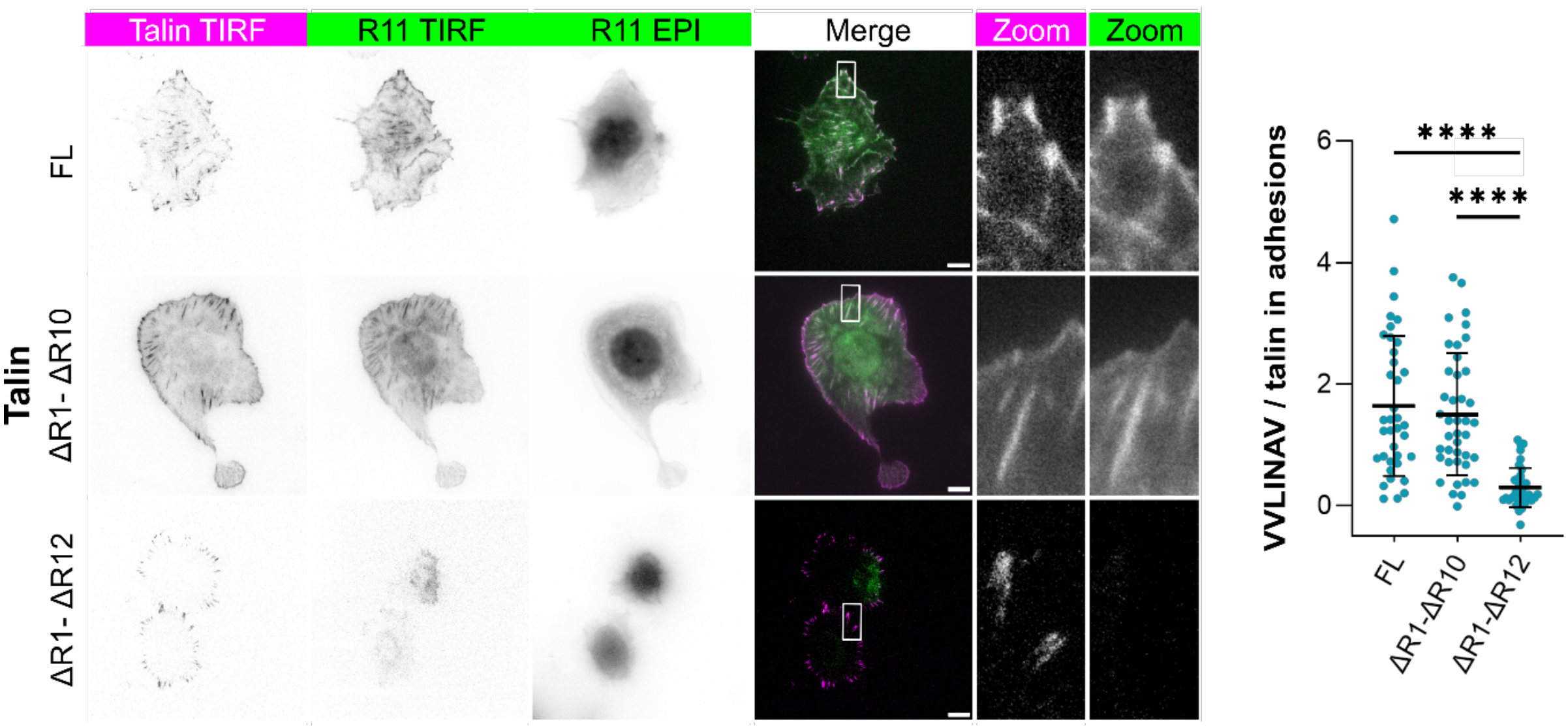

Supplementary Figure 2. **The VVLINAV region of R11 is sufficient to recruit talin to adhesions that contain R11. (A)** Representative TIRF images showing colocalisation of the VVLINAV region with full-length talin and talin lacking domains R1–R10, Talin(ΔR1-R10) but not with talin lacking domains R1-R12, Talin(ΔR1–R12). Scale bar 10 µm. **(B**) Quantification of VVLINAV colocalisation at adhesion sites for the indicated talin constructs (three independent experiments n ≈ 40 cells). Statistical significance was assessed using Brown–Forsythe and Welch ANOVA tests. Data are presented as mean and SD from three independent biological replicates. Statistical significance is indicated as follows: *P < 0.05; **P < 0.01; ****P < 0.0001.

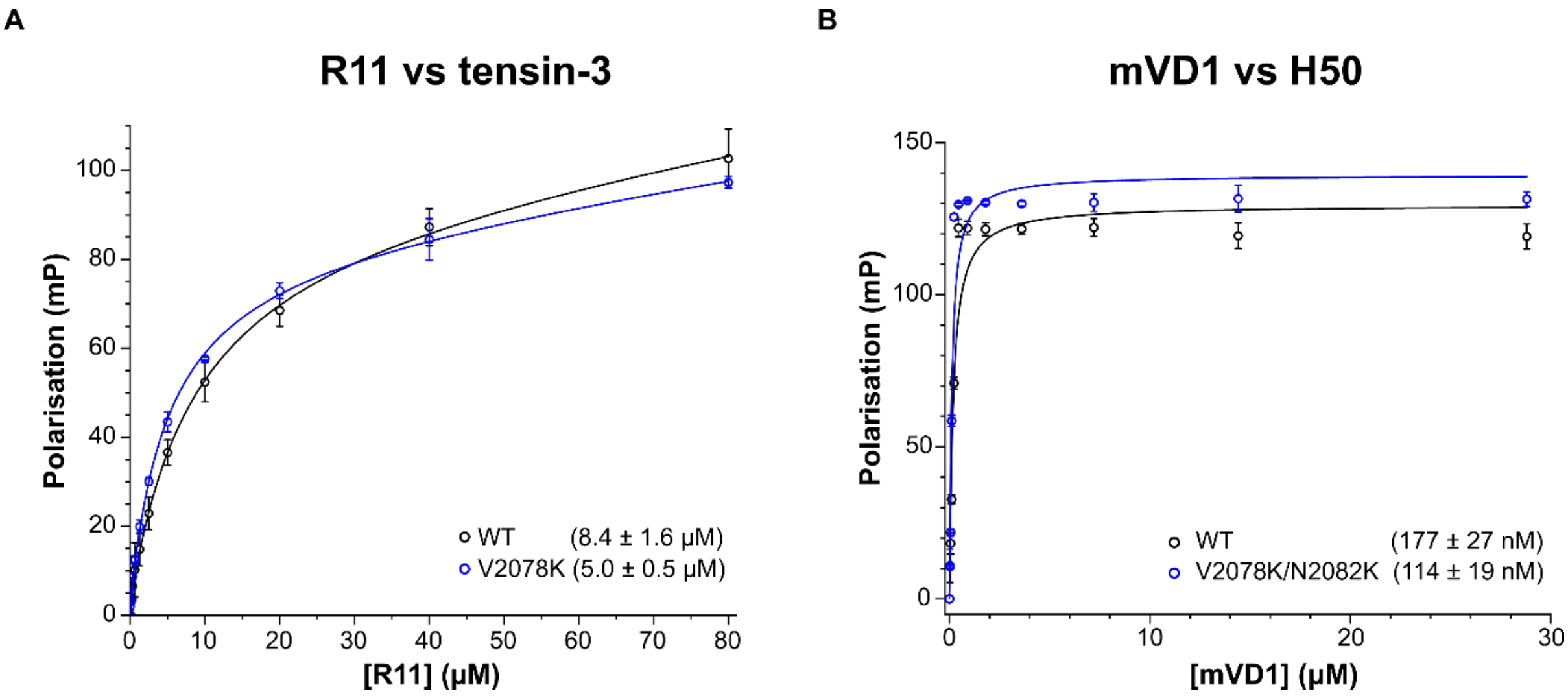

Supplementary Figure 3. **The gatekeeper mutations do not disrupt known R11 interactions.** Fluorescence polarisation assays showing binding of **(A)** WT (black) or V2078K (blue) Tln1(R11) protein to a fluorescein-labelled tensin-3 peptide and **(B)** mVD1 protein to fluorescein-labelled WT (black) or V2078K/N2082K (blue) mTln1 H50 peptides. K_d values were calculated using a single site binding model with non-specific binding and are shown inset with associated standard errors.

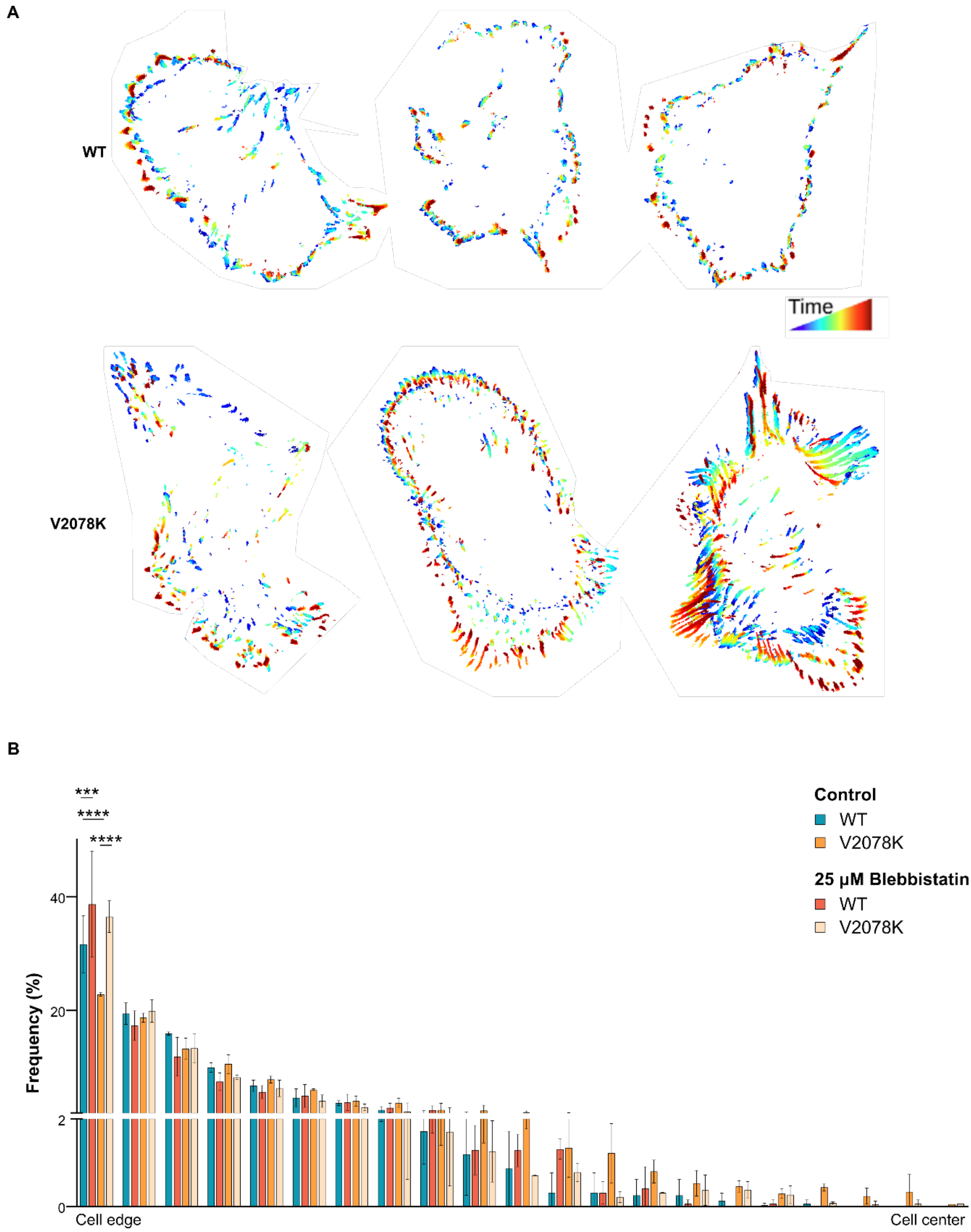

Figure S4. **Analysis of focal adhesion dynamics and their spatial organisation within cells. (A)** Representative FAAS track maps showing the spatiotemporal displacement of focal adhesions during time-lapse imaging. Tracks are colour-coded to indicate adhesion movement over time. **(B)** Quantification of focal adhesion distribution in WT- and V2078K-expressing cells following blebbistatin treatment (25 μM). Statistical significance was determined using two-way ANOVA followed by Tukey's multiple comparisons test. Data are presented as mean ± SD from two independent biological replicates (n = 20 cells). Statistical significance is indicated as follows: *P < 0.05; **P < 0.01; ***P < 0.001; ****P < 0.0001.

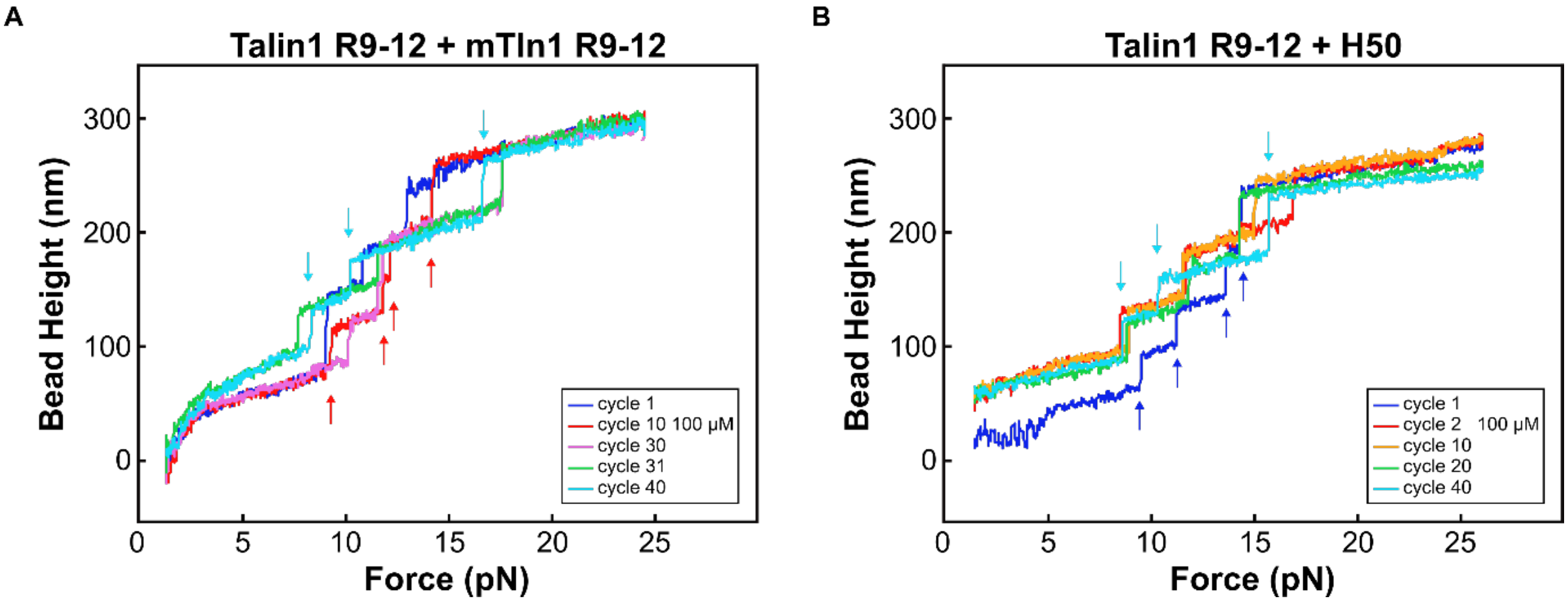

Supplementary Figure 5. **R9-R12 and H50 both bind to mechanically-stretched R9-R12.** Representative consecutive force-height curves obtained from a tether of R9-R12 at a loading rate of 2 ± 0.04 pN/s are shown. **(A)** 100 μM R9-R12 is added as a ligand to the tethered R9-R12 after cycle 10. Within a few cycles one unfolding step is lost. Once lost, this step is lost in all subsequent cycles. **(B)** 100 μM H50 is added as a ligand to the tethered R9-R12, and within a few cycles one unfolding step is lost, once lost this step is lost in all subsequent cycles.

Supplementary Movie

Supplementary Movie 1. **Animated version of Figure 6A showing how vinculin acts as a mechano-chaperone for talin.** When R11 is folded, H50 and the APR are cryptic inside R11 which is able to bind to "0" state binders like tensin-3. Force on talin leads to unfolding of R11 which exposes H50 which can either bind to vinculin, or be extended to a beta-sheet form where it presents the high affinity Aggregation-Prone Region 1 (APR1) which can bind to other talin molecules. Vinculin can resolve these talin multimers by binding to the VBS in H50 demonstrating a novel role of vinculin as a mechano-chaperone for talin.

References

1. C. A. Schneider, W. S. Rasband, K. W. Eliceiri, NIH Image to ImageJ: 25 years of image analysis. *Nat Methods* **9**, 671–675 (2012).

2. E. Meijering, O. Dzyubachyk, I. Smal, "Methods for Cell and Particle Tracking" in *Methods in Enzymology*, (Elsevier, 2012), pp. 183–200.

3. M. E. Berginski, S. M. Gomez, The Focal Adhesion Analysis Server: a web tool for analyzing focal adhesion dynamics. *F1000Res* **2**, 68 (2013).

4. M. Yao, *et al.*, The mechanical response of talin. *Nature Communications* **7**, 11966 (2016).

5. H. Chen, *et al.*, Improved High-Force Magnetic Tweezers for Stretching and Refolding of Proteins and Short DNA. *Biophysical Journal* **100**, 517–523 (2011).

6. X. Zhao, X. Zeng, C. Lu, J. Yan, Studying the mechanical responses of proteins using magnetic tweezers. *Nanotechnology* **28**, 414002 (2017).

7. S. B. Smith, Y. Cui, C. Bustamante, Overstretching B-DNA: the elastic response of individual double-stranded and single-stranded DNA molecules. *Science* **271**, 795–799 (1996).
